# Use of an in silico knowledge discovery approach to determine mechanistic studies of silver nanoparticles-induced toxicity from in vitro to in vivo

**DOI:** 10.1186/s12989-022-00447-0

**Published:** 2022-01-14

**Authors:** Bin-Hsu Mao, Yi-Kai Luo, Bour-Jr Wang, Chun-Wan Chen, Fong-Yu Cheng, Yu-Hsuan Lee, Shian-Jang Yan, Ying-Jan Wang

**Affiliations:** 1 Department of Environmental and Occupational Health, College of Medicine, National Cheng Kung University, No. 1, University Road, Tainan City, 701 Taiwan; 2 Department of Cosmetic Science and Institute of Cosmetic Science, Chia Nan University of Pharmacy and Science, Tainan City, 71710 Taiwan; 3 Department of Occupational and Environmental Medicine, National Cheng Kung University Hospital, Tainan City, 70403 Taiwan; 4 Institute of Labor, Occupational Safety and Health, Ministry of Labor, No. 99, Lane 407, Hengke Road, Sijhih District, New Taipei City, 22143 Taiwan; 5 Department of Chemistry, Chinese Culture University, No. 55, Hwa-Kang Road, Yang-Ming-Shan, Taipei City, 11114 Taiwan; 6 Department of Cosmeceutics, China Medical University, No. 91, Hsueh-Shih Road, Taichung City, 40402 Taiwan; 7 Department of Physiology, College of Medicine, National Cheng Kung University, No. 1, University Road, Tainan City, 701 Taiwan; 8 Department of Medical Research, China Medical University Hospital, China Medical University, No. 91, Hsueh-Shih Road, Taichung City, 40402 Taiwan

**Keywords:** Silver nanoparticles, Knowledge discovery in databases (KDD), Apoptosis, Autophagy, Cell cycle distribution, Pancreatitis

## Abstract

**Background:**

Silver nanoparticles (AgNPs) are considered a double-edged sword that demonstrates beneficial and harmful effects depending on their dimensions and surface coating types. However, mechanistic understanding of the size- and coating-dependent effects of AgNPs in vitro and in vivo remains elusive. We adopted an in silico decision tree-based knowledge-discovery-in-databases process to prioritize the factors affecting the toxic potential of AgNPs, which included exposure dose, cell type and AgNP type (i.e., size and surface coating), and exposure time. This approach also contributed to effective knowledge integration between cell-based phenomenological observations and in vitro/in vivo mechanistic explorations.

**Results:**

The consolidated cell viability assessment results were used to create a tree model for generalizing cytotoxic behavior of the four AgNP types: SCS, LCS, SAS, and LAS. The model ranked the toxicity-related parameters in the following order of importance: exposure dose > cell type > particle size > exposure time ≥ surface coating. Mechanistically, larger AgNPs appeared to provoke greater levels of autophagy in vitro, which occurred during the earlier phase of both subcytotoxic and cytotoxic exposures. Furthermore, apoptosis rather than necrosis majorly accounted for compromised cell survival over the above dosage range. Intriguingly, exposure to non-cytotoxic doses of AgNPs induced G2/M cell cycle arrest and senescence instead. At the organismal level, SCS following a single intraperitoneal injection was found more toxic to BALB/c mice as compared to SAS. Both particles could be deposited in various target organs (e.g., spleen, liver, and kidneys). Morphological observation, along with serum biochemical and histological analyses, indicated that AgNPs could produce pancreatic toxicity, apart from leading to hepatic inflammation.

**Conclusions:**

Our integrated in vitro, in silico, and in vivo study revealed that AgNPs exerted toxicity in dose-, cell/organ type- and particle type-dependent manners. More importantly, a single injection of lethal-dose AgNPs (i.e., SCS and SAS) could incur severe damage to pancreas and raise blood glucose levels at the early phase of exposure.

**Supplementary Information:**

The online version contains supplementary material available at 10.1186/s12989-022-00447-0.

## Background

The rapid advances in nanotechnology in the past decades have provided a myriad of opportunities for mass production of engineered nanomaterials (ENMs) of various chemical types including metals and nonmetals. Among the wide spectrum of currently used ENMs, silver nanoparticles (AgNPs) have attracted considerable popularity in the scientific world due to their excellent antimicrobial effect, electrical conductivity, and catalytic activity [1]. With the ability to tailor particle size and flexibility in surface modification, AgNPs are continuing to find applications in different industrial sectors, such as healthcare, pharmaceuticals, cosmetics, textiles, and electronics. However, the extensive usage of AgNPs-enabled products has raised significant public concerns over their safety issues and environmental impacts. As a matter of fact, multiple lines of in vitro and in vivo studies have indisputably recognized that AgNPs exposure, even at environmentally-relevant doses, can bring about adverse effects mechanistically via dissolved Ag ions and reactive oxygen species (ROS)-dependent pathways [2–4]. In addition to dosage, it appears that physical dimensions and surface chemistry also have important role in determining toxicology of AgNPs [5]. Even so, there is still a lack of study on toxic manifestations and corresponding mechanisms of AgNPs with distinct sizes and surface modifications, especially at the organismal level.

ENMs are typically coated with dispersants of different categories (e.g., surfactants, polymers and reducing agents) during the synthesis processes to stabilize the colloidal particles and prevent them from agglomeration or aggregation in an aqueous solution by increasing repulsive inter-particle forces [6]. Besides, the variety of dispersants also imbues ENMs of the same parent substance with diverse physicochemical properties, particularly with respect to hydrodynamic size and surface potential. As per a systemic appraisal of the scientific documents [7], the top three dispersants employed over the past decades for fabricating AgNPs oriented to common applications were as follows: citrate (27%), polyvinylpyrrolidone (PVP) (18%), and amines (8%). Citrate is loosely adsorbed to the core of AgNPs and thereby stabilizes the colloids by charge repulsion. PVP is a high-molecular-weight polymer; though also non-covalently conjugated to the silver surface, it introduces steric repulsion to ensure greater stability [8]. By contrast, amines (e.g., cysteamine) covalently stabilize AgNPs via formation of Ag-sulfur bonds [9]. Citrate-mediated stabilization renders negative charges to AgNPs surface, while cysteamine-stabilized AgNPs surface appears to be positively charged. In actual fact, differential surface charging attributed to the choice of coating agents has been shown to dictate the modalities of cell death evoked by various ENMs, inclusive of AgNPs [10, 11]. Although the majority of documents have suggested that positively charged AgNPs are more toxic towards eukaryotic cells than negatively charged AgNPs, some conflicting in vivo evidence exists [12, 13]. Accordingly, a more comprehensive study linking both in vitro cytotoxic profiling across multiple cell lines and in vivo systemic toxicity testing is desirable to elucidate how surface coatings contribute to toxicological impacts of AgNPs.

The use of animals for experimental purposes has been subjected to criticism based on both ethical and practical considerations, and these concerns provoke the desire to apply the 3Rs principles (i.e., reduction, refinement, and replacement) in research and develop alternative methods to substitute for traditional anima tests [14]. In light of this, the focus of nanotoxicology research has shifted from phenomenological observations to developing novel in vitro*, *ex vivo*, *in silico*,* and combined strategies for mechanistic explorations; the technological advances also help reduce the use of animals in toxicity testing [15]. Knowledge discover in databases (KDD) is a workflow process that enable identification of valid, novel, useful, and fathomable patterns from large or complicated datasets. This process had been adopted by Horev-Azaria et al. to establish a predictive model for elucidating cytotoxic behavior of cobalt nanoparticles (CoNPs) using small-scale cell viability datasets [16]. They took advantage of a hierarchical tree-structured algorithm to prioritize the toxicologically relevant attributes, including exposure dose, cell type, compound type, and exposure time. The in silico methods provide valuable perspectives that facilitate further planning of any studies translating from in vitro to in vivo.

In this study, we desired to phenotypically and mechanistically explore the toxicity of AgNPs with different dimensions (i.e., smaller-sized v.s. larger-sized) and dissimilar surface coatings (i.e., non-covalent/citrate-coated v.s. covalent/cysteamine-coated) via integration of in vitro, in silico, and in vivo approaches. Apart from the above physico-chemical parameters (hereafter referred to as AgNP type), other cytotoxicity-relevant attributes, embracing exposure dose, cell type, and exposure time, were also taken into consideration so as to obtain a multi-dimensional view of AgNPs-bio interactions from multi-cell-line and computational analyses. By exploiting the in silico prediction results, we prioritized the above attributes and identified a meaningful combination of cell type plus exposure scenarios for further detailed in vitro mechanistic investigations, from the standpoints of cell death modalities (i.e., apoptosis, necrosis, and autophagy), cell cycle distribution, and cellular senescence. Eventually, acute organismal toxicity was assessed by intraperitoneally (IP) injecting female BALB/c mice with a single dose of distinctly coated AgNPs of comparable sizes. In addition to comparing the magnitude of toxicity via survival analysis, we also determined the biodistribution kinetics of the injected AgNPs and examined morphological and histological features of the affected vital organs.

## Results

### Physicochemical characterization of SCS, LCS, SAS, and LAS

Nano-scaled particles, relative to their bulk counterparts, exhibit unique properties, which may not only impart the particles with beneficial characters but also ironically confer distinctive toxicity capacity upon them. In fact, the physicochemical attributes, including size, shape, surface area, zeta potential, agglomeration/aggregation state, and chemical composition, are deemed to chiefly influence their toxic manifestations [17, 18]. For this reason, physicochemical characterization is a recommended prerequisite for implementing nanoparticle toxicity studies. Here, we took advantage of the most widely used techniques (Table 1) to measure several toxicity-related physicochemical parameters belonging to four types of fabricated AgNPs, namely smaller citrate-coated AgNPs (SCS), smaller cysteamine-coated AgNPs (SAS), larger citrate-coated AgNPs (LCS), and larger cysteamine-coated AgNPs (LAS), and to demonstrated that they were all eligible for this study.

**Table 1:** Physico-chemical properties of SCS, LCS, SAS, and LAS as well as their measurements

Items of physico-chemical properties	Measurement	AgNPs			
		SCS	LCS	SAS	LAS
Surface chemistry		Citrate	Citrate	Cysteamine	Cysteamine
Morphology	TEM	Roughly spherical	Roughly spherical	Roughly spherical	Roughly spherical
Actual diameter (nm)	TEM	17.9 ± 1.8	77.3 ± 7.6	19.4 ± 1.2	50.5 ± 4.1
Hydrodynamic diameter (nm) (in water)	DLS	25.5 ± 6.2	81.6 ± 19.6	28.4 ± 6.9	54.2 ± 13.0
Polydispersity (PDI)^a^ (in water)	DLS	0.315 ± 0.021	0.185 ± 0.011	0.307 ± 0.01	0.291 ± 0.06
Hydrodynamic diameter (nm) (in medium)	DLS	193.2 ± 50.1	106.8 ± 26.9	259.9 ± 63.1	78.7 ± 19.4
Polydispersity (PDI) (in medium)	DLS	0.179 ± 0.135	0.229 ± 0.022	0.257 ± 0.005	0.278 ± 0.011
Zeta potential (mV) (in water)	PALS	− 35.8	− 16.7	17.6	19.4
Zeta potential (mV) (in medium)	PALS	− 20.1	− 8.22	− 19.7	− 16.5
Maximum absorbance (nm) (in water)	UV–Vis	391	432	389	430
Maximum absorbance (nm) (in medium)	UV–Vis	403	440	403	431

^a^A polydispersity index (PDI) of 03 and below indicates that the suspension is homogeneous and mono-dispersive

As visualized by electron transmission microscopy (TEM) (Additional file 1: Fig. S1A), both of the citrate- and cysteamine-coated AgNPs, regardless of the particle size, were shown to exhibit roughly spherical morphologies. In terms of actual dimensions, there existed a significant difference between smaller- and larger-sized AgNPs (Table 1), thus enabling us to further explore whether AgNPs-induced toxicity has size dependency. The energy-dispersive X-ray (EDX) microanalysis is performed in conjunction with TEM. It is a technique that detects X-ray emission from a solid surface bombarded with a focused electron beam, and can qualitatively and semi-quantitatively characterize the elemental composition of a specimen. As shown in EDX spectrum images (Additional file 1: Fig. S1B), the TEM specimens of the four AgNP types all had strong signal peaks for elemental carbon and copper, which were the constituents of a TEM grid. A mild signal for elemental sulfur might be reasonably detected in samples of cysteamine-coated AgNPs. Furthermore, a slight amount of elemental silicon was found to be deposited onto some AgNPs-loaded TEM grids; we speculated it as a trace adulterant unexpectedly discharged from the borosilicate glass vials for AgNPs storage. Ruling out carbon, copper, sulfur, and silicon, primary particles of the four AgNP types were exclusively composed of silver; there were no other detectable impurity elements existing in these samples.

The number-weighted size distributions obtained from the DLS analysis showed that the hydrodynamic diameter of any one of the four AgNPs samples, when suspended in pure water (Additional file 1: Fig. S1C), was slightly greater than the primary particle size estimated by TEM. Several studies have implicated that NPs suspended in cell culture media tend to become agglomerated [19–21]. As observed in Additional file 1: Fig. S1D, the culture medium (i.e., DMEM/F12K + 1% FBS) contributed to significant increases in their hydrodynamic dimensions. Intriguingly, this alteration was especially prominent for smaller AgNPs. In terms of particle size distribution, both water- and culture medium-based suspensions of the four AgNP types appeared grossly homogeneous, as their PDI values were found to be either smaller than or approximately equivalent to 0.3 (Table 1).

According to the results of zeta potential measurement (Table 1), citrate coating was shown to provide AgNPs in aqueous suspensions with negatively charged surfaces; AgNPs coated with cysteamine by contrast appeared to be positively charged. Medium-based suspensions just made citrate-coated AgNPs less negatively charged; however, positively charged cysteamine-coated AgNPs were radically converted into negatively charged particles in consequence of being suspended in the cell culture medium. Other qualitative and quantitative physicochemical descriptions of SCS, LCS, SAS, and LAS as well as their measurements are summarized in Table 1.

### Exposures to SCS, LCS, SAS and LAS respectively induced cytotoxicity in both dose-dependent and cell type-specific manners

**Fig. 1:**
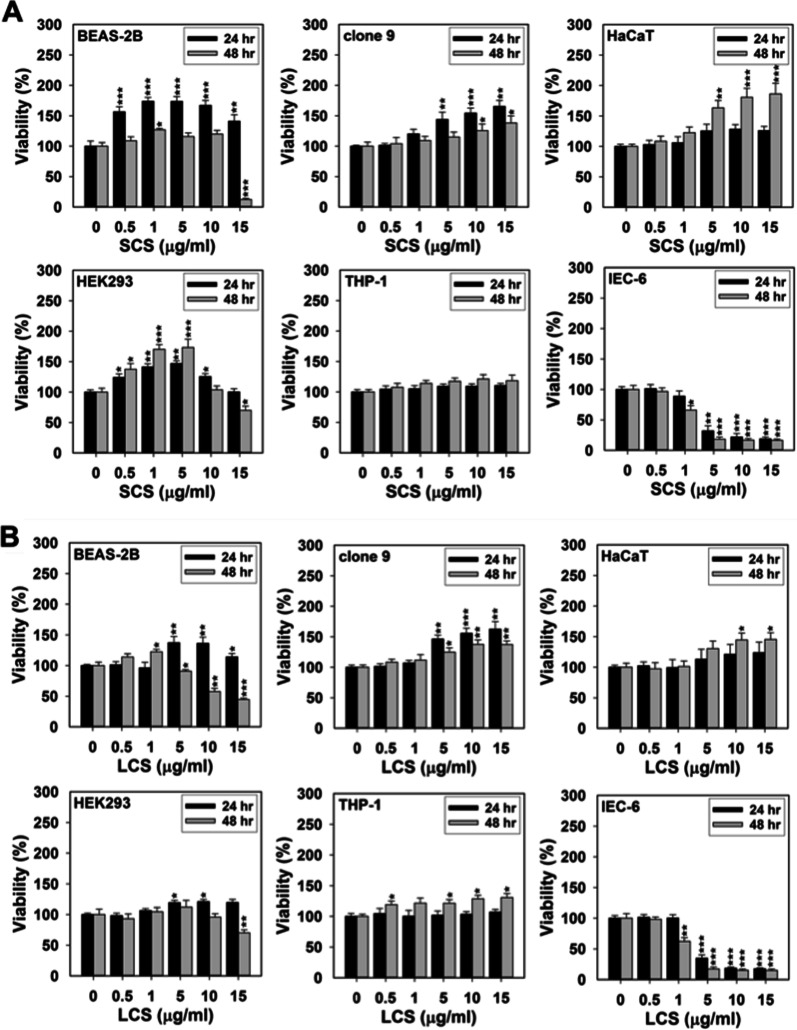

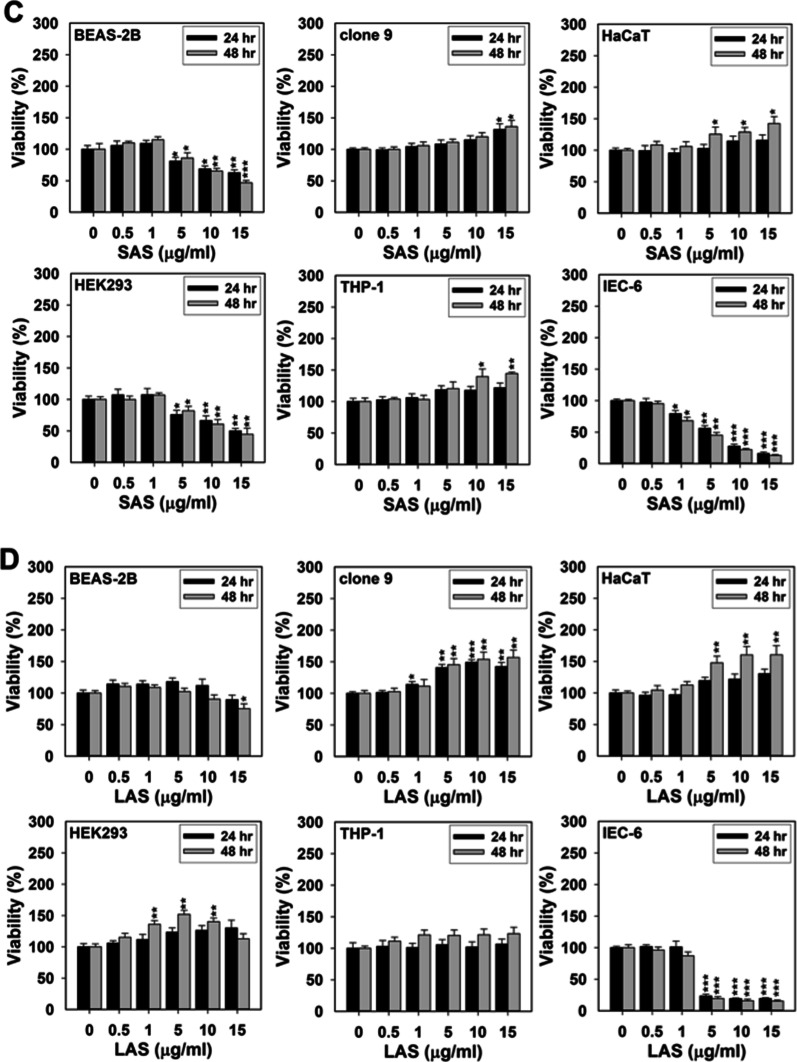
Percent viability of BEAS-2B, clone 9, HaCaT, HEK293, THP-1, and IEC-6 cells affected by respective exposures to **A** SCS, **B** LCS, **C** SAS, and **D** LAS. Each cell type was treated with any of the four AgNP types at the indicated doses (0.5, 1, 5, 10 and 15 µg/ml) for 24 and 48 h, respectively. Relative quantity of viable cells was then quantified by the MTS assay. Results were representative of three independent experiments performed in triplicate. *(*P* < 0.05), **(*P* < 0.01) and *** (*P* < 0.005) denote significant differences between the control (i.e., 0 µg/ml) and treatment groups at the same time point

**Fig. 2:**
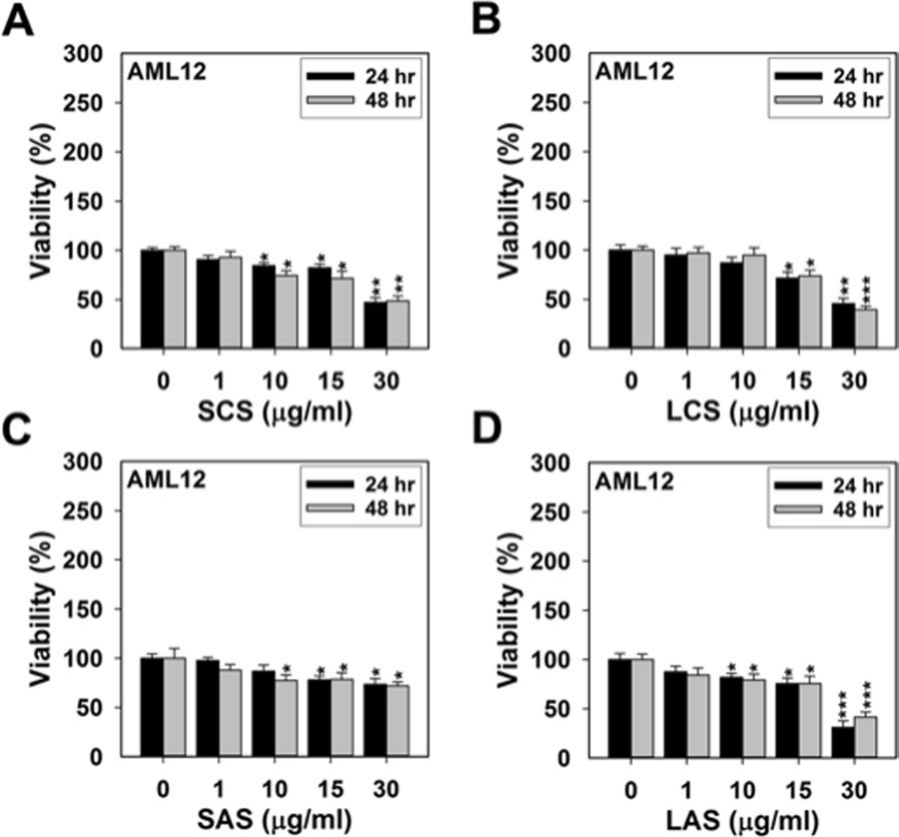
Percent viability of AML12 cells affected by respective exposures to **A** SCS, **B** LCS, **C** SAS, and **D** LAS at the indicated doses (1, 5, 10, 15 and 30 µg/ml) for 24 and 48 h, respectively. Relative quantity of viable cells was then quantified by the MTT assay. Results were representative of three independent experiments performed in triplicate. *(*P* < 0.05), **(*P* < 0.01) and *** (*P* < 0.005) denote significant differences between the control (i.e., 0 µg/ml) and treatment groups at the same time point

Seven mammalian cell lines, including non-tumorigenic human lung epithelial cells (BEAS-2B), normal rat and mouse liver epithelial cells (clone 9 and AML12), immortal human epidermal keratinocytes (HaCaT), human embryonic kidney cells (HEK293), rat small intestinal epithelial cells (IEC-6), and human monocytes (THP-1), were used to represent the potential target organs following AgNPs exposure. We chose to employ either of the two colorimetric tetrazolium-based assays (i.e., 3-(4,5-dimethylthiazol-2-yl)-5-(3-carboxymethoxyphenyl)-2-(4-sulfophenyl)-2H-tetrazolium (MTS) and the 3-(4,5-dimethylthiazol-2-yl)-2,5-diphenyltetrazoliumbromide (MTT)) to detect alteration in viability of the abovementioned cells after exposure to the four AgNPs types. It has been documented that the MTS assay has multiple ideal features of a good assessment system (e.g., ease of use, accuracy, acceptable sensitivity and specificity, and speedy indication of cell viability, proliferation and cytotoxicity) when being applied to in vitro toxicology research [22]. More recently, Liang et al. suggested that employing the MTS assay, compared to the MTT assay or other commonly used methods (e.g., detection of lactate dehydrogenase (LDH) release), could enable minimization of NPs’ interference occurring during the evaluation procedure and ultimately affecting data validity [23]. As AML12 cells were found to be more tolerant of AgNPs challenge in our study, the maximum and minimum values of the exposure dose designated for this cell line (1 and 30 µg/ml) were twice as much as those for the other tested cell lines (0.5 and 15 µg/ml). After 24 h and 48 h of exposure, the dose–response relationships between the four AgNP types and the seven mammalian cell lines are depicted in the separate subpanels of Figs. 1 and 2.

To elaborate the relationships in a more explicit manner, this paragraph begins with interpreting the assessment data of SCS -induced cytotoxicity. At 24 h post-exposure, SCS at several indicated doses was shown to mildly, moderately and even substantially boost growth of BEAS-2B, clone 9, HaCaT, and HEK293 cells (Fig. 1A), suggesting that SCS demonstrate the pro-proliferative potential for these cell lines. Compared to BEAS-2B and Clone 9 cells, SCS exposure allowed HaCaT and HEK293 cells to enter a much more robustly proliferative state within 24–48 h of the exposure. Although the maximum dose (i.e., 15 µg/ml) of SCS enabled a considerable reduction in viability of BEAS-2B and HEK293 cells at 48 h post-treatment (*p* < 0.05), it nevertheless showed pro-proliferative activity towards clone 9 and HEK293 cells, being unable to evoke cell death of either one (Fig. 1A). As a critical component of the innate immune system, we observed that the viability values of THP-1 cells, after given any indicated doses of SCS particles, just became marginally increased during the initial 24 h and slightly greater within 24–48 h (Fig. 1A). Surprisingly, at a relatively low dose (the LC50 value was in the range of 1–5 µg/ml), SCS effectively killed off the IEC-6 cells merely within the initial 24 h of exposure. Clone 9 and AML12 are homologous cells isolated separately from the hepatic tissue of a rat and a mouse. As shown in Fig. 2A, SCS compromised the viability of AML12 cells in both dose- and time-dependent manners. Although the viability of this cell line was profoundly lost upon exposure to 30 µg/ml of SCS, we could still observe an effective viability decline of approx. 30% following exposure to 15 µg/ml of SCS for 48 h. Hence AML12 cells were more susceptible to SCS-induced toxicity than clone 9 cells.

Next, to further clarify the impacts of particle size and coating agent of AgNPs on the dose–response profiles exhibited by the seven cell lines, we followed the descriptive sequence of the above statements and used the toxicity profile of SCS as reference to provide collective elaboration of the toxic behaviors expressed by LCS, SAS and LAS. In addition, the cell viability data depicted in Figs. 1 and 2 were reorganized so as to plot the single-cell-line-versus-multiple-particle-type dose–response graphs, which are more informative in discerning the impact of each AgNP type on viability of a single cell line (Additional file 1: Fig. S2). Taking a panoramic view of the entire viability assessment results (Figs. 1, 2 and Additional file 1: Fig. S2), we found that among these examined cell lines, the overall dose–response trends for LCS- and LAS-induced cytotoxic or pro-proliferative effects are fairly similar to those for SCS-induced effects. During the initial 24 h, exposure to LCS and LAS particles, to a certain degree, also promoted proliferation of BEAS-2B, clone 9, HaCaT, and HEK293 cells in a dose-dependent manner (Fig. 1B, D and Additional file 1: Fig. S2A–D). Comparatively speaking, although exposure to higher doses (i.e. ≥ 10 µg/ml) of either LCS or LAS could also compromise viability of BEAS-2B cells within 24–48 h, such cell line appeared more vulnerable to LCS (because of a more profound reduction in viability ratios). Although longer exposure to 15 µg/ml of LCS appeared cytotoxic to HEK293, exposing this cell line to LAS (1–10 µg/ml) for a similar period conversely led to elevated proliferation (Fig. 1B, D and Additional file 1: Fig. S2D). Of the four AgNP types, SAS seemed to exhibit a distinctive cytotoxic behavior. As shown in Additional file 1: Fig. S2A and D, the viabilities of BEAS2-B and HEK293 cells were effectively curtailed upon exposure to SAS at several indicated doses (≥ 5 µg/ml), whereas they could tolerate toxicity-induced by SCS, LCS, and LAS, and even became proliferative under otherwise similar experimental conditions. Even so, SAS could still be able to enhance proliferation of clone 9 and HaCaT cells as the other AgNP types could be. In reality, all the SAS-exposed cell lines, excepting BEAS-2B and HEK293, had dose–response patterns nearly analogous to those exhibited by SCS, LCS and LAS particles.

As was the case with the borderline pro-proliferative effect on THP-1 cells exerted by SCS, we also observed that the number of such cell line remained largely unchanged or just minorly increased when being challenged with any given doses of SAS, SCS, and LCS particles (Fig. 1A–D and Additional file 1: Fig. S2E). Oppositely, IEC-6 cells among others were extremely vulnerable to any of the four AgNP types; nonetheless, LCS seemed less effective in inhibiting viability of such cell line at the dose equivalent to 5 µg/ml (Additional file 1: Fig. S2F). Even though the survival of AML12 cells could be restricted by exposure to any of the four AgNP types in a dose-dependent fashion, the values of their effective dose fifty (ED 50) were all up to 30 µg/ml, by more than two-fold increase as compared with those used for exposing other susceptible cell lines (Fig. 2 and Additional file 1: Fig. S2G). The cytotoxicity/pro-proliferative activity profiles of the four AgNP types among these cell lines were summarized in Table 2. Overall, we suggest that “cell type”, being secondary to “exposure dose”, should be much more important than “particle size” and “surface coating” in dependency of AgNPs-induced toxicity.

**Table 2:** The summary of cytotoxicity and proliferative activity profiles of SCS, LCS, SAS, and LAS

	SCS	LCS	SAS	LAS
24 h of exposure				
BEAS-2B	Highly pro-proliferative at any given doses	Mildly to moderately pro-proliferative at doses greater than or equal to 5 µg/ml	Moderately toxic at doses greater than or equal to 5 µg/ml	Mildly pro-proliferative at doses less than or equal to 10 µg/ml; Mildly toxic at 15 µg/ml
Clone 9	Moderately to highly pro-proliferative at doses greater than or equal to 5 µg/ml	Highly pro-proliferative at doses greater than or equal to 5 µg/ml	Mildly pro-proliferative at doses greater than or equal to 5 µg/ml	Mildly pro-proliferative at doses less than or equal to 10 µg/ml; Mildly toxic at 15 µg/ml
HaCaT	Moderately pro-proliferative at doses greater than or equal to 5 µg/ml	Mildly to moderately pro-proliferative at doses greater than or equal to 5 µg/ml	Mildly pro-proliferative at doses greater than or equal to 10 µg/ml	Moderately pro-proliferative at doses greater than or equal to 5 µg/ml
HEK293	Moderately to highly pro-proliferative at doses less than or equal to 10 µg/ml	Mildly to moderately pro-proliferative at doses greater than or equal to 1 µg/ml	Moderately to highly toxic at doses greater than or equal to 5 µg/ml	Mildly to moderately pro-proliferative at any given doses
THP-1	Mildly pro-proliferative at any given doses	Mildly pro-proliferative at any given doses	Mildly pro-proliferative at any given doses	Mildly pro-proliferative at any given doses
IEC-6	Highly toxic at doses greater than or equal to 5 µg/ml	Highly toxic at doses greater than or equal to 5 µg/ml	Moderately to highly toxic at doses greater than or equal to 1 µg/ml	Highly toxic at doses greater than or equal to 5 µg/ml
AML12	Mildly toxic at doses less than or equal to 15 µg/ml; Highly toxic at 30 µg/ml	Mildly to moderately toxic at doses less than or equal to 15 µg/ml; Highly toxic at 30 µg/ml	Mildly to moderately toxic at doses less than or equal to 30 µg/ml	Mildly to moderately toxic at doses less than or equal to 15 µg/ml; Highly toxic at 30 µg/ml
48 h of exposure				
BEAS-2B	Mildly to moderately pro-proliferative at doses less than or equal to 10 µg/ml; Highly toxic at 15 µg/ml	Moderately proliferative at doses less than or equal to 1 µg/ml; Moderately to high toxic at doses greater than or equal to 5 µg/ml	Moderately to highly toxic at doses greater than or equal to 5 µg/ml	Mildly pro-proliferative at doses less than or equal to 5 µg/ml; Mildly to moderately toxic at doses greater than or equal to 10 µg/ml
Clone 9	Mildly to moderately pro-proliferative at any given doses	Mildly to moderately pro-proliferative at any given doses	Mildly to moderately pro-proliferative at doses greater than or equal to 5 µg/ml	Highly pro-proliferative at doses greater than or equal to 5 µg/ml
HaCaT	Mildly to moderately pro-proliferative at doses less than or equal to 1 µg/ml; Highly pro-proliferative at doses greater than or equal to 5 µg/ml	Moderately to highly pro-proliferative at doses greater than or equal to 5 µg/ml	Mildly to highly pro-proliferative at any given doses	Highly pro-proliferative at doses greater than or equal to 5 µg/ml
HEK293	Highly pro-proliferative at doses less than or equal to 5 µg/ml; Moderately toxic at 15 µg/ml	Mildly pro-proliferative at doses less than or equal to 5 µg/ml; Moderately toxic at 15 µg/ml	Moderately to highly toxic at doses greater than or equal to 5 µg/ml	Mildly to highly pro-proliferative at any given doses
THP-1	Mildly pro-proliferative at any given doses	Mildly to moderately pro-proliferative at any given doses	Moderately to highly pro-proliferative at doses greater than or equal to 5 µg/ml	Mildly to moderately pro-proliferative at any given doses
IEC-6	Moderately to highly toxic at doses greater than or equal to 1 µg/ml	Moderately to highly toxic at doses greater than or equal to 1 µg/ml	Moderately to highly toxic at doses greater than or equal to 1 µg/ml	Moderately to highly toxic at any given doses
AML12	Mildly to moderately toxic at doses less than or equal to 15 µg/ml; Highly toxic at 30 µg/ml	Mildly to moderately toxic at doses less than or equal to 15 µg/ml; Highly toxic at 30 µg/ml	Mildly to moderately toxic at doses less than or equal to 30 µg/ml	Mildly to moderately toxic at doses less than or equal to 15 µg/ml; Highly toxic at 30 µg/ml

Highly pro-proliferative: viability ≥ 140%; Moderately pro-proliferative: 120% ≤ viability < 140%; Mildly pro-proliferative: 100% ≤ viability < 120%; Highly toxic: viability < 50%; Moderately toxic: 50 ≤ viability < 80%; Mildly toxic: 80% ≤ viability < 100%

### Predictive modeling of AgNPs-induced toxicity using the decision tree-based KDD process

We made use of the in vitro cytotoxicity profiles of the four AgNP types, which were generated from the MTS- or MTT-based cell viability analysis, to create a simple, small-sized nanotoxicity-related database for AgNPs alone. This consolidated database included the following configuration parameters: (i) cell type (i.e., cell line name); (ii) AgNP type; (iii) exposure dose; (iv) exposure time; and (v) the extent of viability reduction. First of all, we followed the KDD methodology, by applying an arbitrary discretization criterion (i.e., a threshold level of viability decrease that is used to define if an effect is toxic or non-toxic to the examined cells), to conduct data transformation, and then adopted the Weka J48 algorithm to undertake the classification analysis, which ultimately built a decision tree for predicting AgNPs-induced toxicity.

**Fig. 3:**
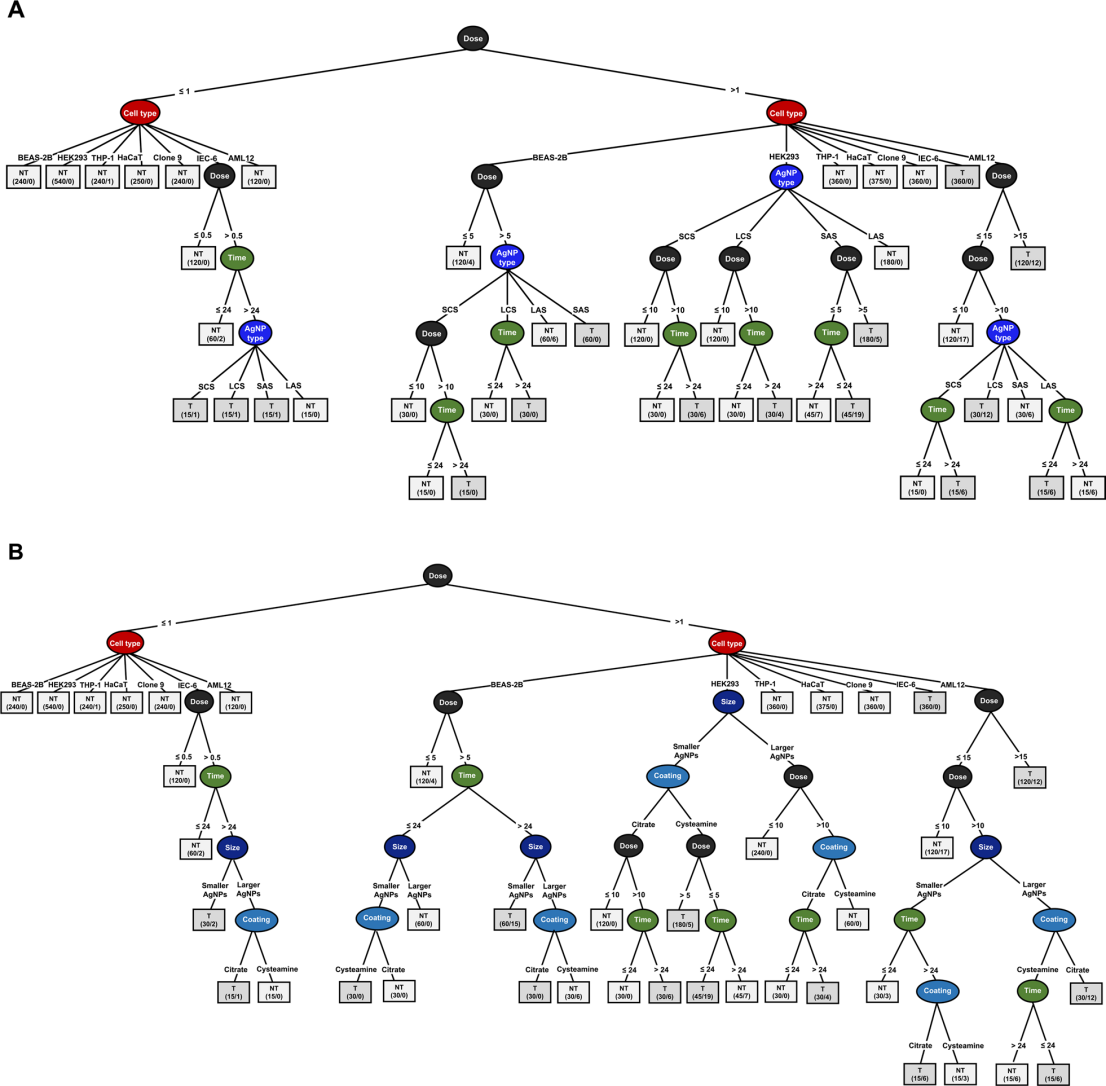
The decision tree model built for predictive ranking of the attributes relevant to assessment of AgNPs-induced toxicity. This learning model was created from a small database consolidating the results of cell viability analyses described in this work. The first decision tree **A** contains four parameters: exposure dose, cell type, AgNP type (SCS, LCS, SAS, and LAS), and exposure time (24 and 48 h) and the second one **B** contains five parameters: exposure dose, cell type, particle size (larger-sized v.s. smaller-sized), surface coatings (citrate v.s. cysteamine). The outcome at each branch terminal is either “nontoxic” (NT-the white square) or “toxic” (T-the gray square), and the numerical data given below the outcome (NT or T) is in the form of n_1_/n_2_, where n_1_ represents the total number of data results (NT or T) and n_2_ represents the number of data results incapable of fulfilling the outcome

In the course of model establishment, having taken into account the measurement error, we used each of the three widely accepted toxicity thresholds, a decrease in cell viability by 20%, 25% and 30%, as the cut-off points to convert the numeric dataset (i.e., percent viability) into the binary (yes/no) data (i.e., toxicity). In this study, we did not aim to digger into the issue with respect to the pro-proliferative activity of AgNPs, and hence regarded it as a “non-toxic effect”. The validity of these cut-off points was verified by comparing the overall accuracy and the sensitivity (as specified by the kappa score) of the given model to any of the above chosen toxicity thresholds. The viability reduction thresholds of 20%, 25% and 30% individually yielded accuracies of 96.97%, 97.28% and 97.08% as well as kappa scores of 0.913, 0.914 and 0.895. Although there was no significant disparity among these accuracies and kappa scores, the decision tree model constructed using the 25% viability reduction as the threshold was finally chosen and shown in Fig. 3A, because of the highest values for the overall accuracy and kappa score.

Moreover, the feasibility of the chosen decision tree model for predicting AgNPs-induced toxicity was also examined by comparing it to that of the renowned “Naïve Bayes model”. At the same cut-off point, the accuracy and kappa score for the model created by the Naïve Bayes classifier were 89.64% and 0.63, respectively, both of which are obviously lower than those of the decision tree model. Table 3 shows the classifier performance metrics of the currently chosen decision tree model. The true positive (TP) rates for the non-toxic class and toxic class are 98.2% and 93.5% respectively, while the false positive (FP) rates for the two classes are merely 6.5% and 0.2%, respectively. Such high-performance measures reassure us about application of the J48 decision tree algorithm to in silico modeling of AgNPs-induced toxicity, and also ensure predictive reliability of the following inferences.

**Table 3:** Performance of the J48 decision tree classifier (evaluated by class) for predictive modeling of AgNPs-induced toxicity

TP Rate	FP Rate	Precision	Recall	F-measure	Class
0.982	0.065	0.984	0.982	0.983	Non-toxic
0.935	0.018	0.972	0.935	0.931	Toxic

TF, True positive; FP, false positive. Detailed information of the J48 decision tree classifier performance was directly obtained from the calculation results of Weka software per se

A so-called decision tree is a tree-structured classification model that provides an intuitive way to interpret the predictions by just following the syntactic if–then-else rule. The attribute at the root node of a decision tree corresponds to the best predictor (i.e., the most dominant attribute or feature) of the selected datasets [16]. As shown in Fig. 3A, in comparison with all the other attributes, “exposure dose” was recognized by the chosen algorithm as the most influential factor that governs the observed toxicity. This model points out that all of the examined cell lines, except IEC-6 cells, could tolerate the cytotoxic effects induced by any of the four AgNP types at doses ≤ 1 µg/ml within 48 h of exposure. IEC-6 cells could exclusively survive exposure to the four AgNP types at doses ≤ 0.5 µg/ml. While this cell line initially remained tolerant to all particle types at doses ranging 0.5–1 µg/ml, exposure to such levels of SCS, LCS, and SAS for a period exceeding 24 h would become harmful. Exposure to AgNPs at doses > 1 µg/ml could cause differential magnitude of cytotoxicity towards BEAS-2B, HEK293, IEC-6 and AML12 cells in a dose-, time-, or AgNP type-dependent manner. However, the four AgNP types at doses ranging 1–15 µg/ml appeared not to be toxic to THP-1, HaCaT and clone 9 cells until the end of exposure. Therefore, it is shown that “cell type” was ranked second by the decision tree algorithm. We suppose that cell type selection is of secondary importance when exploring the toxicity profile of AgNPs.

The attributes located at subsequent internal nodes, from the third rank to the lowest rank, comprise AgNP type, cell type-dependent dose, time, dose-dependent AgNP type, AgNP type-dependent dose, time-dependent AgNP type, AgNP type-dependent time, and dose-dependent time. The order of influential strength of the four toxicity-relevant attributes can be concluded from the model graph (Fig. 3A) and shown as follows: exposure dose > cell type > AgNP type ≥ exposure time. Since “AgNP type” is a dual-domain parameter depicting AgNPs with two dissimilar particle sizes (i.e., smaller v.s. larger) and two disparate surface coatings (i.e., citrate v.s. cysteamine), we reclassified the consolidated datasets, by “particle size” and “surface coating”, to acquire a more in-depth viewpoint pertaining to the ranking of these two attributes on AgNPs-induced cytotoxicity. As shown in Fig. 3B, the order of priority was suggested by the decision tree model to be as follows: exposure dose > cell type > particle size > exposure time ≥ surface coating. Besides, the tree model also suggests the following hierarchy of cell susceptibility towards AgNPs-induced toxicity: IEC-6 ≥ BEAS-2B ≥ HEK-293 > AML12 > Clone-9 = HaCaT = THP-1. Regarding the toxic potential, we can infer from the decision tree diagram that there exist distinct cell type-specific plus dose-dependent ranking patterns for the four AgNP types: (1) SAS = SCS = LCS > LAS (IEC-6; 0.5–1 µg/ml); (2) SAS > LCS > SCS > LAS (BEAS-2B; 5–10 µg/ml); (3) SAS > SCS = LCS > LAS (HEK293; 1–10 µg/ml); and (4) LCS > SCS = LAS > SAS (AML12; > 10 µg/ml).

### Apoptosis served as an underlying mechanism of AgNPs-induced cell death

**Fig. 4:**
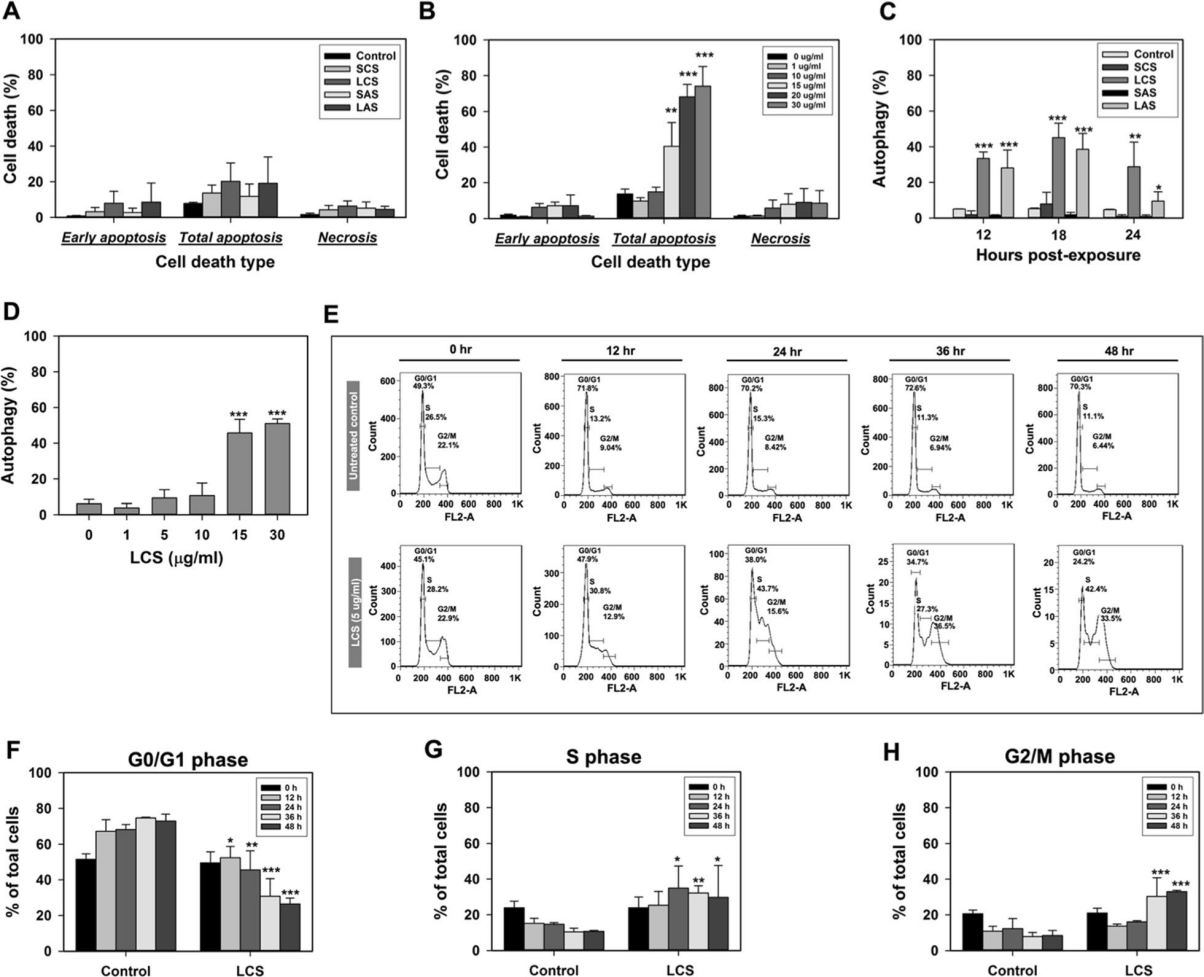
The influences of AgNPs on cell death modalities and cell cycle distribution. **A** The apoptotic and necrotic events occurring in AML12 cells treated with 15 µg/ml of SCS, SAS, LCS, and LAS respectively for 24 h. Total apoptosis represents the sum of the cells undergoing early-stage or late-stage apoptosis over the entire cell population. **B** The dose-dependent response pattern of LCS-evoked apoptosis in AML12 cells. **C** Time course analysis of the autophagic events taking place in AML12 cells treated with the respective one of the four AgNP types (15 µg/ml; 12, 18, and 24 h). **D** The dose- response pattern of LCS-induced autophagy in AML12 cells. **E** Time-course analysis of the impacts of nontoxic-dose LCS exposure (5 µg/ml) on cell cycle phase distribution. The DNA content of the cells was measured by flow cytometry in combination with PI staining. The representative cell cycle histograms for untreated control and LCS-treated AML12 cells are shown as follows: **F** G0/G1 phase, **G** S phase, and **H** G2/M phase. Results were representative of three independent experiments performed in triplicate. *(*P* < 0.05), **(*P* < 0.01) and *** (*P* < 0.005) denote significant differences between the control and treatment groups

Considering the diversity of surface chemical properties, particularly as a result of dissimilar surficial coatings, and their importance in mediating nano-bio interactions, the next parts of this research were to clarify if AgNPs cause particle type-dependent differential effects at dissimilar subcellular levels and to more specifically address the underlying mechanisms. According to the results of the above viability assessments and decision analysis, we decided to choose the cell line AML12 as the in vitro model for subsequent mechanistic experiments. Though showing detectable decreases in viability upon treatment with any of the four AgNP types (≥ 15 µg/ml), AML12 cells were less vulnerable to AgNPs-induced toxicity than other cell lines used in this study. To discern which one of the cell death modalities (i.e., apoptosis and necrosis) has a primary role in AgNPs-induced cytotoxicity and if the particle type matters much to the identified event, AML12 cells following 24 h of respective exposures to 15 µg/ml of SCS, LCS, SAS, and LAS, were stained with Annexin V-FITC and PI and then analyzed by flow cytometry. As seen in Fig. 4A, all except SAS were shown to induce apoptosis, the percentages of which all appeared somewhat greater than that of the basal apoptosis detected in the untreated cells. By contrast, neither of the four AgNP types seemed to be able to elicit necrotic cell death, since its levels were quite comparable to that of the untreated control.

To further explore this observed phenomenon, we chose to make use of LCS, which by comparison stimulated a relatively greater magnitude of apoptosis, to determine the dose–response pattern. As shown in Fig. 4B, exposure to serial doses of LCS (1, 10, 15, 20 and 30 µg/ml) enabled occurrence of apoptosis in a dose-dependent way, but it did not coordinately enhance the magnitude of necrosis. An analogous dose–response patterns of apoptosis and necrosis were also observed in IEC-6 cells treated with serial doses of SAS (Additional file 1: Fig. S3A–B). Taken together, these data suggest AgNPs-induced apoptosis as a predominant mechanism accounting for decreased cell viability, though its magnitude also depends on cell type and particle type.

### Autophagy was an early event of AgNPs-induced cytotoxicity

Multiple lines of evidence have shown that various nanomaterials, including AgNPs, can potently activate autophagy both in vitro and in vivo [2, 3, 24–27]. Most recently, it has been suggested that autophagy, as it is a basic stress response and a regulator of various subcellular events, is able to act as a toxicity biomarker-like indicator applicable to NPs safety assessments in pharmaceutical, food, and cosmetic industry sectors [28]. In this section, we continued exploring if particle type dependency modulates AgNPs-evoked autophagic activity. Seeing that autophagy is considered an early pro-survival response under stress conditions, two additional earlier time points (i.e., 12 h and 18 h), apart from 24 h, were included in the experiment to monitor its dynamic changes within the exposed cells.

A flow cytometric analysis of acridine orange-stained cells revealed that unexposed AML12 cells maintained consistently low levels of basal autophagy at each time point (Fig. 4C). These data indicated that smaller-sized AgNPs (i.e., SCS and SAS) at 15 µg/ml were unable to elicit autophagy in AML12 cells. On the contrary, larger-sized AgNPs (i.e., LCS and LAS) at the equivalent dose could remarkably stimulate a time-dependent increase in autophagic activity, which lasted at least until 18 h after the exposure and then appeared to gradually attenuate. By comparison, LCS evoked greater responses in these events, and as such it was subjected to further dose–response analysis. A dose–response trend plus a dramatic upsurge at 15 µg/ml was detected at 18 h post-exposure (Fig. 4D). Altogether, the above results suggest that AgNPs, depending on particle size and surface coating, display differential pro-autophagic competency.

In order to clarify whether cell susceptibility also dictates the autophagic outcome induced by AgNPs, we also used the highly susceptible IEC-6 cells to conduct similar exposure assessments. The results showed that SCS, LCS, and LAS at 5 µg/ml enabled significant increases in autophagic activities at 8 h post-exposure, whereas an equal dose of SAS just led to marginally elevated autophagic activity (Additional file 1: Fig. S3C). Even so, SAS could still lead to dose-dependent activation of autophagy in IEC-6 cells (Additional file 1: Fig. S3D). In terms of the dynamic alteration in autophagic activity induced by serial doses of SAS, we found that all except 0.5 µg/ml could maximize the activity at 8 h post-exposure. The autophagic activity of each dose group was dramatically declined within 8–18 h after exposure, approaching to the basal autophagic level (Additional file 1: Fig. S3D). Taken together, these results suggest that AgNPs can enable activation of autophagy in dose-dependent, cell type-specific, and/or particle type-related manners. Besides, as an early event, exposure time is also critical for detecting AgNPs-induced autophagic activity.

### AgNPs at non-cytotoxic doses had the potential to induce a G2/M arrested senescent status

In light of the inferences from the above decision tree analysis, one interesting thing we found is that exposure time was, with respect to the cytotoxicity profile of AgNPs, ranked as the least influential attribute among others. Besides, the viability assessments indicated that the survival statuses of AML12 cells, at 24 h and 48 h after exposure to any of the four AgNP types, respectively, were in relatively equal proportions. Thus, we conjectured that cellular protective actions, such as the above-mentioned autophagy and “cell cycle arrest”, would be undertaken by the exposed cells to mitigate AgNPs-induced cytotoxicity. It has been noted previously in several in vitro studies that cells exposed to sublethal and lethal doses of AgNPs shall undergo G2/M cell cycle arrest, which accounts for commitment to apoptosis [29–31]. Nevertheless, whether AgNPs at the no-observed-cytotoxic-effect level affect cell cycle distribution is yet to be clarified. To address it, we chose to expose the AML12 cells to 5 µg/ml LCS, which might just cause very minor to undetectable levels of cell death within 48 h, and evaluated the influence of such exposure on cell cycle distribution at every 12 h interval (from 0 to 48 h).

The cell cycle graphs and the quantitative results (Fig. 4E–H) revealed that the unexposed cells underwent G0/G1 phase cell cycle arrest beyond 24 h of culture as a consequence of contact inhibition. By contrast, 5 µg/ml LCS significantly reduced the percentage of AML12 cells in the G0/G1 cell cycle phase at 12 h post-exposure while it also concurrently and profoundly increased that of the S-phase cells. There existed a time-dependent trend towards a sustained decrease and increase in percentages of the cells respectively residing at the G0/G1 and S phases. Lastly, it resulted in accumulation of cells in G2/M phase, which peaked at roughly 36 h post-exposure. These results suggest that exposure to AgNPs even at doses unable to cause cell death still can enable cell cycle arrest at the G2/M phase.

A growing body of evidence has suggested that persistent growth arrest at the G2/M phase has a vital role in inducing senescence [32]. Since AML12 cells, as stated above, were shown to undergo G2/M cell cycle arrest after prolonged exposure to 5 µg/ml LCS, we surmised that these cells would likely become committed to senescence. To examine our assumption, we conducted cytochemical determination of senescence-associated β-galactosidase (SA-β-gal) activity, a widely accepted hallmark of senescent cells. Under bright-field microscopy (Additional file 1: Fig. S4A), greenish blue staining that represents SA-β-gal positive cells was absent among the unexposed AML12 cells after 48 h of culture. On the contrary, SA-β-gal positive cells could be observed among the cells exposed to no-observed-cytotoxic-effect levels of LCS (i.e., 1, 5 and 10 µg/ml), albeit there was great variability in the degree of staining among the positive cells. In short, these data suggest that cellular senescence most likely takes place among low-dose AgNPs-exposed cells. As senescent cells display a reduced proliferation ability compared to younger cells, we then further evaluated the clonogenic potential of the AML12 cells following 48 h of prolonged exposure to 1 and 5 µg/ml of LCS. As can be seen in Additional file 1: Fig. S4B–C, 1 µg/ml LCS could marginally reduce the number of the resulting colonies on the 7th day after the pronged exposure, whereas 5 µg/ml LCS brought about a considerable reduction. Taken together, prolonged exposure of the cells to low-dose AgNPs might make them unable to undergo cell division, which is a consequence of G2/M arrested senescence.

### AgNPs exposure differentially triggered apoptosis, autophagy and cell cycle arrest in a dose-dependent manner

On the basis of the aforementioned in vitro findings, we came up with an assumed notion for explicating how cells react to and defend against the cytotoxic effects elicited by differential doses of AgNPs: On one hand, autophagic machinery is activated upon a higher-dose AgNPs exposure so as to efficiently eliminate the protein aggregates and injured organelles resulting from the internalized particles. However, it has been shown that AgNPs exposure contributes to autophagy dysfunction, which might further evoke apoptosis in both direct and indirect fashions [2]. On the other hand, cells receiving a lower-dose challenge may suffer DNA damage and enter a state of cell cycle arrest, which is a self-protective event that alleviates cellular destruction by allowing time for DNA repair to occur and by avoiding propagation of damaged DNA to daughter cells. Basically, cells can resume cell-cycle progression after escaping from genotoxic stress [33]. Nevertheless, DNA repair capacity of certain individual cells, owing to sub-clonal heterogeneity within a cell line, may appear more likely to be overwhelmed by AgNPs-induced damage; these cells finally commit to irreversible cell cycle arrest and become senescent. To validate this assumption, we determined time-course expression levels of the proteins involved in regulation of apoptosis, autophagy, and cell cycle progression in AML12 cells after exposure to a noncytotoxic and a subcytotoxic dose of LCS (i.e., 5 µg/ml and 15 µg/ml), respectively.

**Fig. 5:**
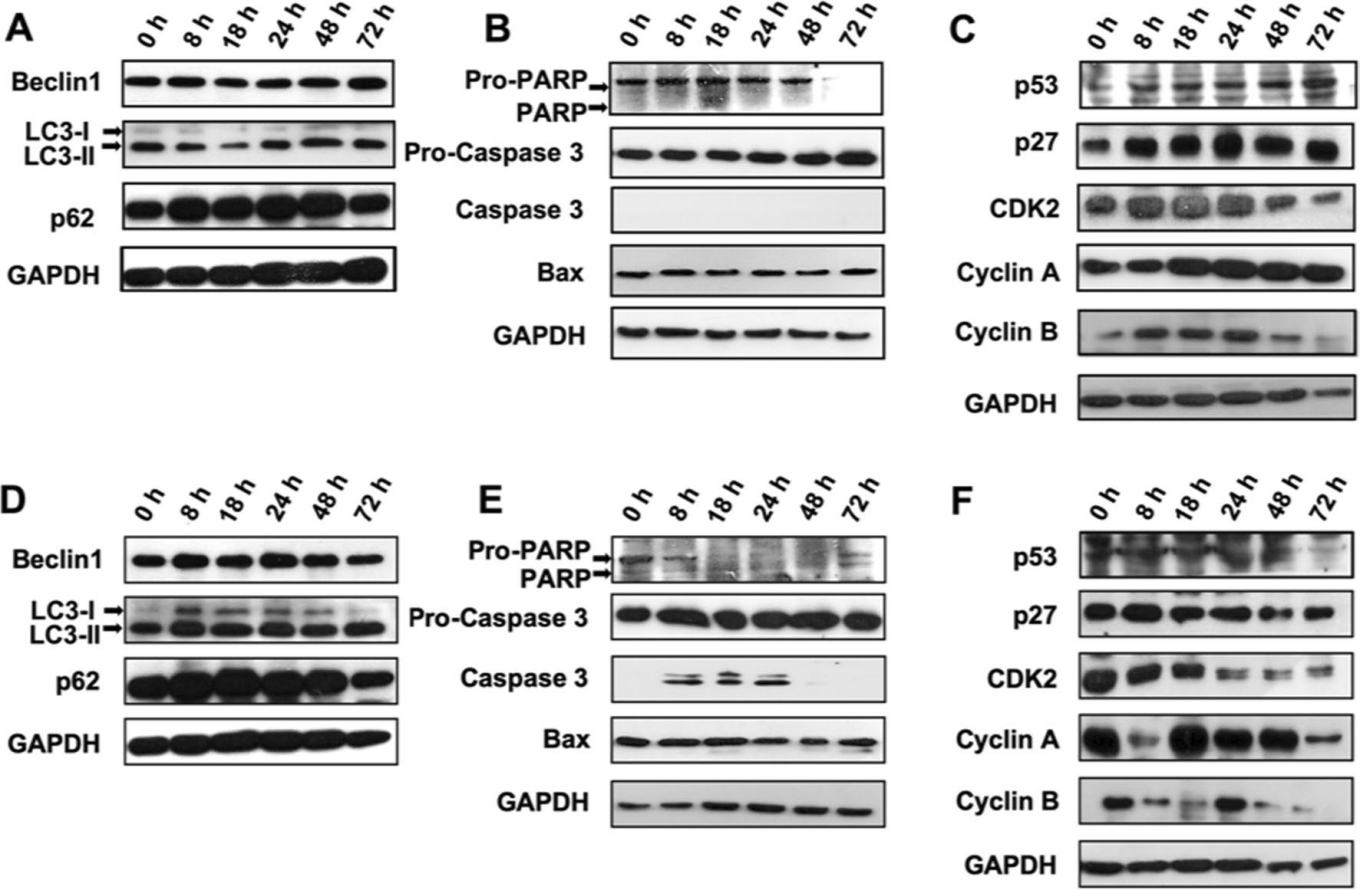
Time-course analysis of the expression levels of autophagy-, apoptosis-, and cell cycle regulation-related proteins in AML12 cells respectively treated with nontoxic and subcytotoxic LCS (i.e., 5 µg/ml and 15 µg/ml). **A** Autophagy-related markers (5 µg/ml); **B** Apoptosis-related markers (5 µg/ml); **C** Cell cycle regulators (5 µg/ml); **D** Autophagy-related markers (15 µg/ml); **E** Apoptosis-related markers (15 µg/ml); and **F** Cell cycle regulators (15 µg/ml). GAPDH was served as a total protein loading control

Upon a noncytotoxic-dose exposure, Beclin 1, a key regulator of autophagy, was shown to be expressed at comparable basal levels at the initial measurement time points (Fig. 5A), but its expression level gradually increased beyond 48 h post-exposure. Similarly, it also took about 24–48 h to elevate the conversion of autophagy marker microtubule-associated protein 1 light chain 3 (LC3) (i.e., from LC3-I to LC3-II). By contrast, the level of p62 appeared to be upregulated within the first 8 h, followed by a maintenance period of approximately 48 h, and then was declined at 72 h post-exposure. It has been noted that p62 is a stress-inducible protein able to sense the redox status of cellular conditions [34]. Hence these data indicate that LCS at noncytotoxic doses still enable induction of oxidative stress, while autophagy, in this case, should be considered a later-stage event. However, exposure to such a dose of LCS didn’t trigger cleavage of both pro-poly (adenosine diphosphate ribose) polymerase (pro-PARP) and pro-caspase 3 at all time points. Moreover, there was also no significant difference in the Bax expression level between different time points (Fig. 5B). As a result, these data confirmed that apoptosis was unable to be triggered in AML12 cells by LCS at doses lower than 10 µg/ml (Fig. 4B), even after a prolonged exposure time.

With respect to cell cycle regulation under noncytotoxic-dose LCS stimulation, the level of p53, a sensor of multiple forms of cellular stress, displayed a time-dependent increasing trend, which started soon after the exposure (Fig. 5C). The increase of p53 was shown to be accompanied with upregulation of p27, which has been proven to be able to negatively affect cell cycle progression [35–37]. Mechanistically, the transition between different cell cycle phases is mainly controlled by checkpoints, which consist of two protein families: cyclins and cyclin-dependent kinases (CDKs). It has already been documented that p27 can induce both G0/G1-phase and G2/M-phase cell cycle arrest via binding to CDK2-cyclin A/E complexes and inhibiting their activities [38–40]. In this study, we observed that accumulation of the p27-CdK2-cyclin E complex (designated as “CDK2” in Fig. 5C) was elevated along with the increased expression of p27 occurring within the first 24 h. However, it gradually reduced after p27 reached a steady-state level. The p27-Cdk2-cyclin A complex (designated as “Cyclin A”) began accumulating beyond the initial 8 h, and then remained at a constant level until 72 h after exposure.

Plenty of evidence has suggested that CDK1 in complex with cyclin B is important for controlling entry into and exit from mitosis [41, 42]. Previous work has identified the involvement of p21, a potent CDK inhibitor, in G2/M checkpoint control. Besides, it has been reported that transient induction of p53 at the G2 phase triggers the onset of G2/M-arrested senescence via p21-mediated inhibition and nuclear retention of Cdk1-cyclin B [43]. As indicated by Fig. 5C, p21 upregulation might be also accompanied by enhanced p53 expression, thereby leading to nuclear accumulation of inactive Cdk1-cyclin B complexes. Undeniably, stable accumulation of the inactive p21-Cdk1-cycelin B (designated as “Cyclin B”) was detected within the initial exposure period, but such accumulation gradually declined in excess of 24 h. Collectively, the results shown in Fig. 5C reflect the likelihood that cells exposed to 5 µg/ml of LCS could undergo cell cycle arrest both in G0/G1 and G2/M phases within 24 h, and for some reason certain of these cells might then irreversibly become senescent. In addition, most of the exposed cells might ultimately resume the cell cycle progression, because of the gradual decline in accumulation of “CDK2” (i.e., p27-CdK2-cyclin E) and “Cyclin B” (i.e., p21-Cdk1-cycelin B). Hence it can be suggested that cells may take advantage of “cell cycle arrest” as a defensive strategy against minor AgNPs exposure.

By contrast, a subtoxic dose (i.e., 15 µg/ml) of LCS appeared to activate autophagy immediately after the exposure, as discernable upregulation of Beclin 1 and p62 as well as a noticeable increase in LC3-I/LC3-II conversion had already emerged at 8 h post-exposure (Fig. 5D). At this dose, the exposed cells retained an effective autophagic clearance capacity, thereby displaying a time-dependent decrease in the amount of p62 beyond 24 h of exposure. In the aspect of apoptosis, these cells already demonstrated elevated levels of Caspase-3 activation, PARP cleavage and Bax expression right at 8 h post-exposure, which indicated that apart from autophagy, apoptosis also occurred soon after such exposure (Fig. 5E). Intriguingly, some cells might eventually survive this challenge, since we observed that the above apoptotic markers became attenuated and even completely disappeared at 48 h and 72 h post-exposure. Altogether, these results suggest that intact autophagy shall be activated by LCS (at least at 15 µg/ml) in the early period of exposure to counteract AgNPs-induced cytotoxicity.

With regard to cell cycle progression, the exposed cells exhibited a marginal rise in expression of p53 and p27 at the 8th hour post-exposure, and appeared to steadily maintain their expression levels until 24 h post-exposure (Fig. 5F). Then, there was a decreasing trend in overall p53 and p27 expression. Furthermore, such higher-dose exposure evoked time-dependent decline in the level of “CDK2” accumulation. On the whole, the levels of accumulated “Cyclin A” and “Cyclin B” within these exposed cells indeed exhibited similar dynamic patterns. Nevertheless, merely at 18 and 24 h post-exposure respectively could be found the exposed cells exhibiting somewhat greater levels of “Cyclin A” and “Cyclin B”, as compared to those of the unexposed cells. Thus, “cell cycle arrest” was not considered a major hallmark of higher-dose LCS exposure. Taken together, these results suggest that autophagy activation is an early pro-survival strategy against toxic-dose AgNPs exposure.

### Exposure to AgNPs via intraperitoneal (IP) injection caused acute toxicity and displayed differential organ distribution and accumulation

The decision tree analysis suggested that particle type, in terms of its influence over AgNPs cytotoxicity profiling, should be considered less important than exposure dose and cell type. However, this attribute does indeed matter, since there have been lots of research showing that size and surface coating affect manifestations of AgNPs-induced toxicity as well as tissue distribution and accumulation in vivo [44–49]. From another perspective, the choice of animal species or even strain also likely produces variations in NPs fate and behavior observed in living systems [49–52]. To mechanistically dissect AgNPs-evoked acute toxicity in vivo and discern the impacts of different particle types on the identical toxicity endpoints, it would be better to employ an established, sensitive and reliable animal model. In the recent past, Cho et al. have shown that IP injection of smaller citrate-coated AgNPs induced a higher degree of acute toxicity in BALB/c mice, compared to their larger-sized counterparts [53]. Hence it was decided to use this strain as the in vivo model for comparing the acute toxic effects exerted by a single IP injection of likewise smaller AgNPs but being separately coated with citrate and cysteamine (i.e., SCS and SAS).

**Fig. 6:**
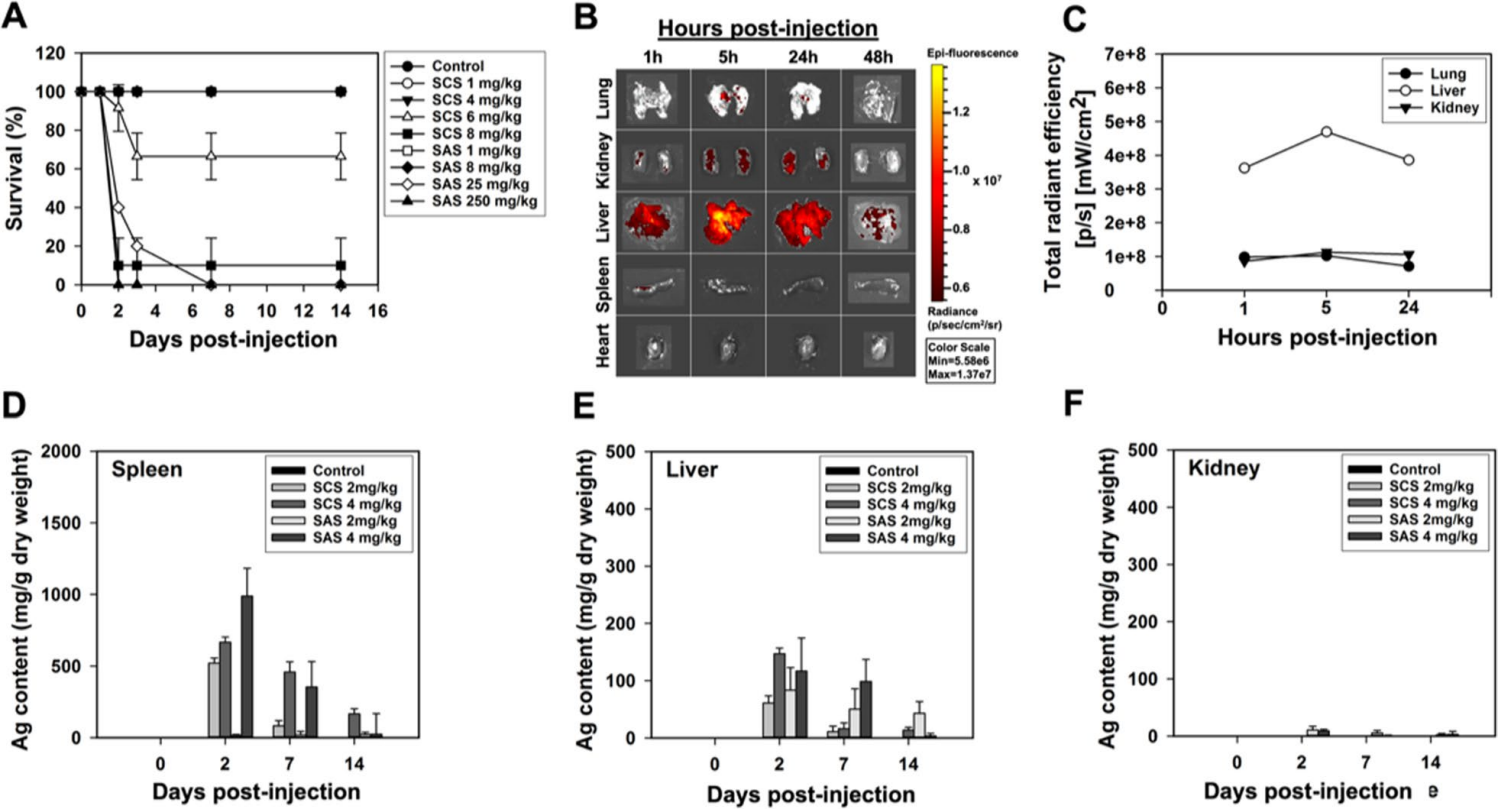
The survival status of AgNPs-treated mice and the distribution/accumulation kinetics of intraperitoneally injected AgNPs during the 14-day observation period. **A** The survival rate of BALB/c mice (n = 3–5 per group) receiving treatment with a single IP injection of different doses of SCS (i.e., 1, 4, 6 and 8 mg/kg) and SAS (i.e., 1, 8, 25 and 250 mg/kg), respectively. **B** Whole-organ IVIS images of lung, kidneys, liver and spleen from mice treated with RBITC-AgNPs (8 mg/kg) for 1, 5, 24 and 48 h. **C** Quantification of IVIS imaging data. The amount of Ag deposited in spleen (**D**), liver (**E**), and kidney (**F**) of SCS- or SAS- treated mice (2 and 4 mg/kg) (n = 3) was determined by GFAAS on days 0, 2, 7, and 14 post-exposure. Mice of the control group (n = 3) in each experiment was treated with a single IP injection of saline

Within the 14-day observation period of the acute toxicity testing (Fig. 6A), the mortality of the mice injected with 6 mg/kg of SCS was shown to approach 40% on the 3rd day after the exposure. When its dose was raised to 8 mg/kg, more than 80% of the exposed animals died in the same length of time. Nevertheless, a single equivalent dose (i.e., 8 mg/kg) of SAS could not kill any of the animals even until the end of observation. SAS could exhibit obvious toxicity towards BALB/c mice, with a dramatic increase in 3-day mortality, only when its dose increased to 25 mg/kg (n = 3–5 mice per group, data combined from three independent experiments). On the whole, these data suggest that cysteamine-mediated stabilization can enable attenuation of AgNPs toxic potential to a certain degree, thereby rendering them higher biocompatibility as compared to the less unstable citrate-coated AgNPs.

To track tissue distribution of the IP injected AgNPs, rhodamine B isothiocyanate (RBITC), a red-emitting fluorophore, was conjugated onto AgNPs surface, and served as a tracer for monitoring dissemination of AgNPs within a living organism. As shown by the imaging results obtained using the IVIS SpectrumCT (Fig. 6B), a considerable quantity of AgNPs was instantly distributed into liver and then very likely taken up by hepatocytes and liver-resident cells at 1 h post-exposure to 8 mg/kg of RBITC-AgNPs. Although the intensity scales of fluorescent signals detected in kidneys and lungs were much weaker than that measured in liver (Fig. 6C), it was worth pointing out that the injected RBITC-AgNPs also had a tendency towards renal and pulmonary distribution and accumulation during the initial 24-h exposure period. Afterwards, such cumulative fluorescent signals appeared to gradually reduce with time, but this phenomenon might be indicative of destabilization of conjugation between RBITC and AgNPs, rather than clearance of AgNPs, in a physiological context. A feebler signal could be identified in spleen at the end of the first hour after injection, yet it might soon decline and appeared utterly undetectable at the fifth hour. We found it rather intriguing that there existed no fluorescent signals in heart at any of the observation time points.

We further assessed the pronged accumulation of AgNPs, or more correctly silver deposition, in the target organs mentioned above, including liver, kidney and spleen, all of which are considered metabolically active. The mice were IP injected with indicated non-toxic doses (i.e., 2 and 4 mg/kg) of SCS and SAS, respectively, and then sacrificed to obtain the organs at 2, 7, and 14 days post-exposure (n = 3 per group). As revealed by Fig. 6D–E, a great amount of silver was found to be deposited both in liver and spleen of SCS- and SAS-treated mice in a dose-dependent manner on the 2nd day after injection. To our surprise, both SCS and SAS particles appeared to more preferentially accumulate in spleen, with an approximately 5–tenfold increase in amount of deposited silver as compared to that detected in liver. Subsequently, the levels of silver deposition in the spleen and liver were shown to dwindle down progressively over time. Relatively speaking, it seemed not easy for SCS and SAS particles to accumulate in kidneys after IP injection even if they might be initially disseminated to this target organ (Fig. 6F). By inference, AgNPs deposited in the metabolically active organs are capable of being removed through certain built-in clearance mechanisms.

### Acute exposure to toxic doses of AgNPs caused profound pancreatic injury

The serum biochemistry profile (Table 4) showed a significantly dramatic increase in magnitude of alanine aminotransferase (ALT) and aspartate aminotransferase (AST) activities in mice having exposed to 8 mg/kg of SCS for 24 h. The increment suggests that hepatitis and acute liver failure occur in response to such exposure. On the contrary, an equivalent dose of SAS didn’t enhance the activity of these two enzymes. Speaking of the renal function, short-term exposure to SCS at such toxic dose didn’t elevate the levels of serum creatinine (CRE) and blood urea nitrogen (BUN). Nonetheless, 8 mg/kg of SAS, though incapable of causing lethality, still led to a detectable increase in levels of CRE and BUN respectively by around 40% and 50%, as compared to untreated mice. It is noteworthy that the activity of serum amylase (AMYL), a biochemical marker for the diagnosis of acute pancreatitis, was significantly upregulated upon exposure to SCS. Besides, there was also an increase, by up to 50%, in the serum glucose level among SCS-exposed mice. According to such serum biochemical profiling data, we speculated that pancreatic injury, aside from hepatitis, might also be a prominent manifestation of AgNPs-induced acute toxicity.

**Table 4:** Serum biochemical data of the control and SCS- and SAS-treated mice

Parameters	Reference	Control	SCS (8 mg/kg)	SAS (8 mg/kg)
ALT (UI/L)	53.00 ± 7.88	38.66 ± 15.14	700.00 ± 262.71*	31.33 ± 5.13
AST (UI/L)	122.01 ± 23.70	192.00 ± 9.16	1198.00 ± 510.65*	221.00 ± 86.74
CRE (mg/dL)	0.34 ± 0.08	0.37 ± 0.05	0.33 ± 0.05	0.53 ± 0.11
BUN (mg/dL)	30.07 ± 9.85	16.63 ± 2.57	20.43 ± 1.33	39.90 ± 13.32
GLU (mg/dL)	151.77 ± 58.63	80.66 ± 22.27	123.33 ± 9.07	63.00 ± 4.58
AMYL (UI/L)	500–1500	1357.00 ± 114.50	5410.66 ± 1483.72**	1301.33 ± 383.91

The reference values for each parameter in female BALB/c mice were provided by the Laboratory Animal Center of NCKU. Data are expressed as mean ± standard deviation. *(*P* < 0.05) and **(*P* < 0.01) denote significant differences between the control and treatment groups. ALT: Alanine aminotransferase; AST: aspartate aminotransferase; CRE: creatinine; BUN: blood urea nitrogen; GLU: glucose; AMYL: amylase

**Fig. 7:**
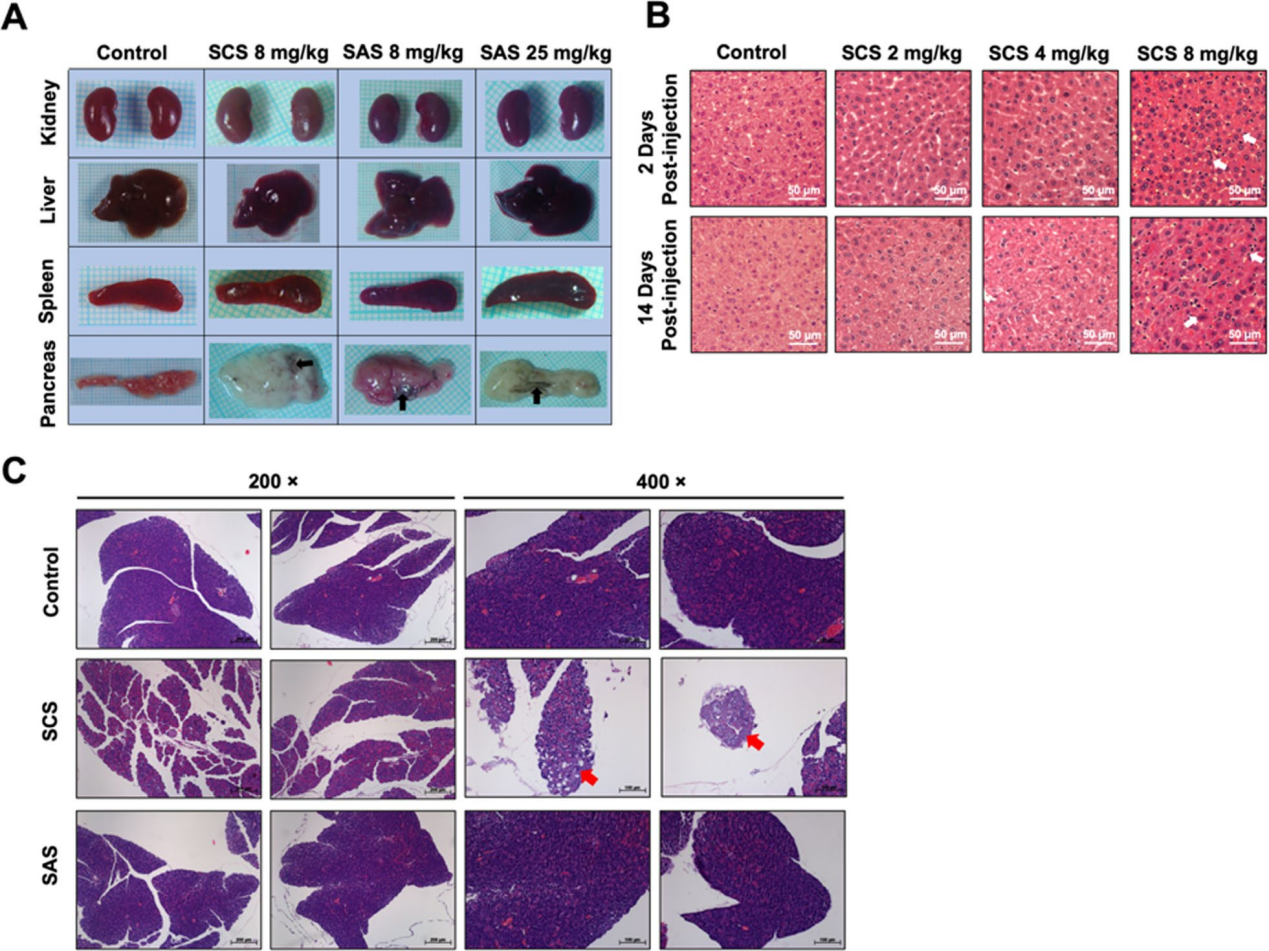
Morphological appearance and histopathology of the major target organs for AgNPs accumulation. **A** Direct observation of morphological alterations in kidneys, liver, spleen, and pancreas from control and SCS- or SAS-treated mice (SCS: 8 mg/kg; SAS: 8 and 25 mg/kg). The black arrow indicates AgNPs accumulation in pancreas. **B** H&E-stained of liver sections from untreated control and SCS- or SAS-treated mice (2, 4 and 8 mg/kg; a single IP injection). Mice were sacrificed to obtain liver tissues on the 2nd and 14th day after exposure. **C** H&E-stained histological sections of pancreas from control and SCS- or SAS-treated mice (8 mg/kg; a single IP injection). Mice were sacrificed to collect pancreatic tissues on the 2nd post-exposure. The red arrow indicates focal necrotic injury in pancreatic tissues of SCS-treated mice

To address this assumption, we further investigated the morphological appearances of liver, kidneys, spleen, and pancreas dissected from mice exposed to 8 mg/kg of SCS and SAS, respectively. On top of that, the observations also included the organ samples from mice subjected to lethal-dose SAS exposure (25 mg/kg). As can be seen in Fig. 7A, irrespective of the AgNP types, there were no observable damages to the outward appearances of liver, kidneys and spleen at 24 h after IP injection. It deserves noting that the pancreas of both SCS- and SAS-treated mice was shown to undergo strikingly distinctive pathological changes within 24 h after IP injection. Normally, the pancreas is an elongated gland with pinkish color, as seen in the untreated control. However, AgNPs exposure resulted in the collapse of the pancreatic structure, and the seriousness of damage was dose-dependently increased. At the lethal dose (SCS: 8 mg/kg; SAS: 25 mg/kg), such exposures completely “whitened” the pancreas, suggesting occurrence of acute hemorrhagic pancreatitis. Notably, a dark diffuse plaque-like deposit could also be identified within the collapsed pancreatic tissues. Altogether, these results implicate that pancreas might be more susceptible to AgNPs-induced organ toxicity, as compared to liver, kidneys and spleen having been described previously [47, 54, 55].

Further confirmation by histopathological analyses (i.e., H&E staining) revealed that SCS at non-toxic doses (2 and 4 mg/kg) seemed to have no essential influence over the liver structure at the microscopic level even until 14 days after exposure. However, a single toxic dose (8 mg/kg) of SCS given to this animal was shown to induce massive immune cell infiltration in the hepatic interstitial space, yet it appeared not to destroy the normal liver structure by the 14th day post-exposure (Fig. 7B). Paraffin-embedded pancreatic sections by contrast showed the existence of severely disintegrated lobular pancreatic structure within 48 h following exposure to 8 mg/kg of SCS. Furthermore, swollen and vacuolized acinar cells, which indicate necrotizing pancreatitis, were also present in the tissue fragments seen in Fig. 7C. Nonetheless, there were no apparently disintegrated fragments and necrotic lesions in pancreatic tissue sections from mice injected with an equivalent dose of SAS. In light of the results of our current study, we strongly urged that pancreatitis is a noticeable concern of AgNPs-induced acute toxicity.

## Discussion

Extensive application of AgNPs and other nanomaterials in our daily life has raised the risks to humans from external or internal exposure to such highly bioactive entities. In light of this, it is imperative for scientists to seek a more thorough understanding of their hazardous effects and potential mechanisms via investigations at different levels- in vitro, in vivo, an even in silico. In this research, we incorporated the in vitro toxicity profiles of four AgNP types (i.e., SCS, SAS, LCS, and LAS), which were obtained from MTT- or MTS-based viability assessments of seven different mammalian cell lines, to establish a small-scale database consisting of five configuration parameters: cell type, AgNP type, exposure dose, exposure time, and cell viability reduction extent (the first four of which are considered relevant to nanotoxicity assessments). We took advantage of a KDD-based methodology, by submitting the above datasets to Weka J48 decision tree classification, to generalize AgNPs toxic behaviors toward the examined cell lines in an in silico manner. This process also evaluated the predictive strength of the above-mentioned toxicity-relevant attributes; they are ranked, as inferred from decision tree model graph (Fig. 3), in the following order: exposure dose > cell type > AgNP type ≥ exposure time. What’s more, hierarchy of cell susceptibility is suggested as follows: IEC-6 ≥ BEAS-2B ≥ HEK-293 > AML12 > Clone-9 = HaCaT = THP-1. At the in vitro level, we further demonstrated that both apoptosis and autophagy occurred soon after higher (subtoxic)-dose AgNPs exposure (Fig. 4C, Additional file 1: Figs. S3B–C and 5D–E). Cellular demise resulting from AgNPs exposure was referable to apoptosis rather than necrosis (Fig. 4B and Additional file 1: Fig. S3A). In this cellular scenario, autophagy per se should be considered a protective mechanism that enables alleviation of AgNPs-induced cellular damage. Nevertheless, at the doses unable to evoke cell death, AgNPs might be also likely to affect the exposed cells, making them undergo cell cycle arrest (Fig. 4E–H and Additional file 1: Fig. 5C). A small minority of the exposed cells even became senescent following such challenge (Additional file 1: Fig. S4A). At the in vivo level, we observed that cysteamine-based functionalization, as compared with citrate-mediated surface stabilization, could largely enhance the biocompatibility of smaller AgNPs (Fig. 6A). An approx. threefold increase from the lethal dose (8 µg/ml) of SCS was needed to bring about a significant reduction in survival of SAS-exposed mice. Being analogous to previous studies [47, 54, 55], our results also showed that spleen, liver, and kidneys were the major target organs of AgNPs distribution and accumulation (Fig. 6B–C). However, as suggested by serum biochemistry analysis and macroscopic examination of the internal organs (Table 4 and Fig. 7A), we then realized that pancreas appeared to be the most significant target organ, being much more vulnerable than others to AgNPs-induced acute toxicity.

To date, growing concepts in animal welfare and ethics have aroused the attention of scientists to embed the 3Rs principles into animal research planning and execution. Synchronously, alternatives to animal testing, including in vitro and in silico approaches, are suggested to reduce or even replace the use of experimental animals in nanotoxicity assessments. Traditional cytotoxicity assessment, as compared to animal testing, is less expensive and faster, but it avoids the ethical issues at the expense of lower predictive values [56]. Advanced 3D cell culture, 2D co-culture, and tissue-engineered systems have been developed to overcome the limitations of conventional cell line-based studies and acquire more predictive data. In silico modeling, a fairly new scientific discipline that combines experimental and computational approaches, presents another option to predict the human health risks of NPs and provides a powerful basis for understanding of biological mechanisms at atomic and molecular levels [14]. Since 2006, the Working Party on Manufactured Nanomaterials (WPMN) established by the Organization for Economic Cooperation and Development (OECD) has been working to develop proper strategies that ensure the safer use of nanomaterials and prevent the potential risks of nanotoxicity. Over the past decades, many modeling approaches have been developed, and certain of them (e.g., nano-QSAR) can enable identification of important factors in affecting NPs toxicity. Several publicly available databases, such as NanoDatabank, NanoHub, NanoMILE, and ModNanoTox, have been established as the source for nanomaterial risk assessments [14]. Although KDD is a process typically accepted for identifying valid, novel, useful, and understandable patterns from large and complex databases, it can also be applied to small data sets as was the case of Horev-Azaria et al.’s research on predictive toxicity modeling of cobalt-based NPs [16]. Therefore, we followed Horev-Azaria et al*.*’s method, adopting the KDD process, together with the J48 decision tree algorithm, to build a prediction model of AgNPs toxicity via analyzing the viability profile of seven mammalian cell lines in response to SCS, LCS, SAS, and LAS particles. The use of decision tree models to describe research findings offers some advantages over other classification algorithms, which have been enumerated in earlier literatures [57, 58]. It’s worth mentioning that the decision tree algorithm can generate simple if–then–else statements to make the subsets at each node purer than the original dataset, and so that investigators can intuitively explain the resulting model without any prior knowledge of data mining (DM) techniques. The idiosyncratic feature of the tree model (Fig. 3) resides in its ability of providing multi-layered perspectives unable to be acquired from conventional visual examination of 2D or even 3D scatter plots, as done in most previous research works.

In this research, we took advantage of the above in silico modeling method to prioritize the toxicity-relevant attributes for our further in vitro and in vivo mechanistic toxicological studies. We confirmed that the J48 decision tree algorithm-based KDD process was feasible for predictive modeling of nanotoxicity, simply based on small-scale datasets as was the case of Horev-Azaria et al.’s investigation [16]. However, the database used by either of these in silico studies were generated from cell culture-based experiments, which fail to closely mimic the relevant in vivo situations. As *Drosophila melanogaster* (fruitfly) and *Caenorhabditis elegans* (nematode) have been recognized as two powerful model systems that can be used to explore the molecular and cellular basis of ENMs-induced toxicity on both organismal and population scales [59, 60]. Because various toxicity endpoints (e.g., mortality, developmental deficits, locomotor performance, lifespan and healthspan, and oxidative stress and gene expression levels) can feasibly be examined in vivo using these two models, we can theoretically establish more informative and comprehensive databases on nano-bio interactions and identify stronger predictors for real-world/in vivo nanotoxicity assessments.

The physicochemical aspects of NPs, particularly with respect to primary particle size and surface charge, have been suggested to have great influence on the mechanisms of their uptake into cells and induced cell death [10]. Indeed, many lines of evidence have pointed out that AgNPs can induce apoptosis via extrinsic and intrinsic pathways and also act as a potent activator of autophagy [2, 3, 24, 61]. In general, smaller-sized or positively charged AgNPs are acknowledged to possess more potent apoptosis- and autophagy-inducing activities than those with bigger dimensions or negative charges [11, 62–64]. Nonetheless, this is not always the case. In 2016, Mishra et al. dissected the cytotoxic mechanisms of PVP-coated AgNPs (particle size: approx. 10, 50 and 100 nm) in human liver-derived hepatoma G2 (HepG2) cells [65]. At 24 h post-exposure, the smaller the particle size, the greater was the strength of apoptosis induced by AgNPs at the subcytotoxic dose (5 µg/ml) (10 nm > 50 nm > 100 nm). On the contrary, AgNPs at cytotoxic doses (10 and 50 µg/ml) reversed the order of the above strengths (100 nm > 50 nm > 10 nm). Moreover, the autophagic flux could be activated when HepG2 cells were treated with AgNPs at the noncytotoxic dose (1 µg/ml) (the strength order: 10 nm > 50 nm > 100 nm). The autophagic activity resulting from exposure to a cytotoxic dose of 10 nm-AgNPs (10 µg/ml) was time-dependently enhanced in the first 12 h, but then it seemed to gradually weaken with time. In our study, though cysteamine-conjugated AgNPs (i.e., SAS and LAS) appeared to be positively charged in aqueous suspensions, they turned negatively charged when suspended in culture media, displaying analogous zeta potential values as citrate-coated AgNPs (i.e., SCS and LCS) did (Table 1). As per the results of the decision tree-based KDD analysis, we chose AML12 cells to go on further mechanistic studies. Intriguingly, irrespective of the coating agents used, we found that larger-sized AgNPs (i.e., LCS and LAS) were more toxic towards AML12 cells, while also showing greater tendency to trigger autophagy and apoptosis (Fig. 2, Additional file 1: Figs. S2G, S4A and C).

As stated in the background section, the release of silver cations owing to oxidative dissolution is considered to underlie the mechanistic basis behind AgNPs toxicity. In 2013, van Aerle et al*.* identified that transcriptomic alterations in zebrafish embryos exposed to AgNPs and their bulk and ionic counterparts were largely similar across all treatments; thus, they suggested that AgNPs toxicity be associated with bioavailability of silver ions at the surface of the particles or those dissolved in the water [66]. The rate and degree of AgNP dissolution depend on physicochemical and biological properties of the simulated fluids (e.g., ionic strength, pH, chemical composition, and amount of protein) [67, 68]. Furthermore, the types of surface coatings can also affect the release of soluble Ag [69, 70]. We had previously examined the dissolution extent of citrate-, cysteamine-, and polyvinylpyrrolidone (PVP)-capped AgNPs respectively incubated in the cell culture medium for 24, 48, and 72 h (our unpublished data). Comparatively speaking, citrate-capped particles exhibited somewhat greater dissolution rates than the other two counterparts, suggesting that cysteamine- and PVP-based functionalization could effectively reduce oxidative dissolution of AgNPs. Regardless of the particle size and surface coating, the concentration of the dissolved silver ions, however, was much lower than the minimal cytotoxic dose of ionic silver alone. As a result, the influence of such AgNP dissolution on viability of the examined cell lines is not taken into consideration in the current study. As a matter of fact, recent research has emphasized that the release of silver ions in the cytosol (i.e., intracellular dissolution), due to degradation of endocytosed AgNPs in the lysosome, demonstrates a far greater impact upon cell damages [71].

Nowadays AgNPs released from nano-enabled consumer products can access the human body via the following routes: dermal absorption, ingestion, or inhalation, which depends on their intended use (e.g., as the antibacterial component of wound dressings, textiles and cosmetics, packaging films and food containers, water disinfectants, and hygiene sprays). Nonetheless, there is an emerging trend in biomedical research toward development of AgNPs-based drugs or drug carriers, and this suggests that such nano-scaled entities might purposely gain direct entry to the human circulatory system via intravenous (IV), intraperitoneal (IP), or subcutaneous (SC) injection [72]. To make use of AgNPs securely in nanomedicine, further advancement of knowledge pertaining to their biodistribution/bioaccumulation, biocompatibility, and manifestations of systemic toxicity is required. In fact, apart from exposure pathways, the toxicological behaviors of AgNPs in vivo are also a function of particle size and surface characteristics (e.g., charge, hydrophilicity, lipophilicity, and catalytic activity) as well as the species or the strain of laboratory animals selected for research [44–52]. In terms of the particle size, Cho et al.’s study demonstrated that female BALB/c mice intraperitoneally injected with 10 nm citrate-coated AgNPs (~ 10 mg/kg) were found dead or moribund within 24 h, whereas those exposed to an equivalent dose of 60 nm and 100 nm AgNPs respectively didn’t show any signs of intoxication. At 6 h post-injection, apparent pathological alterations were observed in liver, spleen, and thymus cortex of the mice exposed to 10 nm AgNPs, while such alterations were not evident following exposure to either 60 or 100 nm AgNPs [53]. More recently, Al-Doaiss et al*.* investigated chronic toxicity of low-dose exposure to five distinct sizes of AgNPs (10, 20, 40, 60 and 100 nm) in male BALB/c mice receiving multiple daily IP injection (1 mg/kg b.w.) [73]. Their findings also indicated that smaller AgNPs would be more toxic than larger counterparts; IP injected AgNPs could evoke prominent histological and histochemical alterations in hepatic, renal and testicular tissues.

Indeed, the choice of the administration route of a given agent should depend on whether researchers intend to examine its local or systemic (either enteral [through the digestive tract] or parenteral [outside the digestive tract]) effects. Administration of substances via the parenteral routes typically produces the highest bioavailability because it avoids the first-pass effect that commonly occurs in the gastrointestinal tract whenever the substances are orally administered [74]. The intraperitoneal (IP) route of administration is particularly useful in acute systemic toxicity studies conducted in rodents, and it is often preferred over the intravenous (IV) route owing to its ease of administration [75]. Besides, IP administration results in faster and more complete absorption as compared to the oral and SC routes. It is worth noting that the pharmacokinetics of substances administered intraperitoneally are more comparable to those observed following oral administration, as mesenteric lymphatic vessels are the primary route for absorption from the peritoneum [76]. Therefore, in this research, we mechanistically explored the acute toxic effects caused by a single IP injection of smaller-sized AgNPs with two distinct surface coating ligands, citrate and cysteamine (i.e., SCS and SAS) respectively, in female BALB/c mice. Although surface modification of NPs with biocompatible agents is an important strategy to render them biocompatible and attenuate NPs-induced toxicity [77], our data still recommend that AgNPs per se should be considered toxic to living organisms despite showing improved biocompatibility. As long as the exposure dose increases to a fairly high extent, the so-called biocompatible AgNPs (SAS in this research), after entry into the animal body, might be also deadly and enable occurrence of systemic adverse events similar to those caused by bio-incompatible AgNPs (e.g., SCS) (Figs. 6A and 7A).

As has been noted before, IP-administered small particles or macromolecules tend to reach systemic circulation via peritoneal lymphatics and are eventually distributed throughout the body [78]. Whole-organ IVIS imaging showed that RBITC/fluorophore-conjugated AgNPs were disseminated into liver, kidneys, lungs, and spleen as soon as after IP injection (roughly within 1–5 h), whereas no fluorescent signal was detected in the heart during this period (Fig. 6B). These results suggest that AgNPs can be efficiently adsorbed into the bloodstream from the peritoneal cavity, and then distributed into various vital and non-vital organs where they may accumulate over time. Given that the liver’s main job is to detoxify xenobiotics, an even more profound degree of fluorescence retention could be observed in the hepatic tissue of RBITC-AgNPs-treated mice (Fig. 6B–C), suggesting that a quantity of injected AgNPs might be distributed to liver and taken up by hepatocytes for detoxification. Even so, serum biochemistry and histopathological analyses showed that hepatic retention of AgNPs may compromise liver function and trigger acute hepatic inflammation (Table 4 and Fig. 7B). Furthermore, quantitative detection of Ag deposition suggests that the amount of AgNPs deposited in the animal body can gradually decline with time, which is possibly due to macrophages-mediated clearance and/or other elimination processes [79, 80]. The most valuable finding in our study is that exposure to SCS (8 mg/kg) and SAS (8 and 25 mg/kg) via the IP route can cause severe damage to pancreas and raise blood glucose levels (Fig. 7A and Table 4). More recently, Tiwari et al*.* found that perinatal AgNPs exposure through mother mice could bring about pancreatic beta-cell death, reduced insulin level, and increased blood glucose levels in their offspring [81]. Apart from AgNPs, it has been demonstrated that repeated oral exposure to zinc oxide (ZnO) NPs evoked chronic pancreatitis in Sprague–Dawley rats [82]. As diabetes mellitus is the major late sequelae of chronic pancreatitis, the results of our research, along with aforementioned former findings, may associate exposure to metallic NPs (e.g., AgNPs and ZnO NPs) with pancreatogenic diabetes. Paradoxically, accumulating evidence has suggested that functionalization of these NPs with some molecules or combined with drugs may conversely alter their properties and demonstrate antidiabetic properties [83]. In reality, numerous nanoparticles have been regarded as double-edged sword; whether they are harmful or beneficial depends on the fabrication process, dosimetry, administration route, type of functionalization, physico-chemical properties, and so on. To address these sophisticated issues, further research combining in vitro, in silico, and in vivo experimental strategies would be desirable.

## Conclusions

The in silico decision tree-based KDD process could be successfully applied to identify attributes relevant to assessment of AgNPs-induced toxicity. This integrated research revealed that AgNPs could exert toxicity in dose-, cell/organ type- as well as particle type-dependent manners both in vitro and in vivo. More importantly, a single IP injection of lethal-dose AgNPs (i.e., SCS and SAS) could incur severe damage to pancreas (pancreatitis) around 24 h after administration. Detailed mechanisms in regard to AgNPs-induced pancreatitis have yet to be interrogated in the future.

## Methods

### Cell cultures

Seven different cell culture models were used: BEAS-2B, clone 9, HaCaT, HEK293, IEC-6, THP-1, and AML12. Table S1 summarizes details of the culture media used for routine maintenance of these mammalian cell lines. In general, the cells were grown at 37 °C in a humidified incubator with 5% CO_2_, and the culture media were replenished every 2–3 days. At 85–95% confluency, the adherent cells were passaged by dislodging with 0.05% trypsin-0.02% EDTA solution, washing with PBS, and seeding in 10 cm dishes at appropriate densities (cells/cm^2^), depending on the cell types. In the AgNPs exposure experiments, the culture protocol consisted of seeding cells in 6-, 12- or 96-well plates or 6 cm dishes, allowing 24 h of incubation for cell attachment and proliferation, and replacing the culture media with fresh ones containing the treatments.

### Apoptosis assay by flow cytometry

As previously reported, apoptotic cell death was assessed by a flow cytometry-based approach using the Annexin V FITC Apoptosis Detection kit (Calbiochem, San Diego, CA, USA) [29]. Briefly, single cell suspensions were prepared by centrifuging the trypsinized cells at 3000 rpm for 5 min, and then resuspending the pellets in 100 µl of 1 X Annexin V-binding buffer (10 mM HEPES, pH7.4, 0.14 M NaCl, and 2.5 mM CaCl_2_) that contained 5 µl of Annexin V-FITC alone or in combination with 10 µl of propidium iodide (PI) solution (50 µg/ml) (Sigma-Aldrich, St. Louis, MQ, USA). After incubation at room temperature for 15 min, 400 µl of 1 X binding buffer was added to terminate the reaction, and then the stained cells were analyzed by flow cytometry using the FACSCalibur cell analyzer with CellQuest™ Pro software (BD Biosciences, Heidelberg, Germany).

### Autophagy assay by flow cytometry

Development of acidic vesicular organelles (AVOs), which are regarded as a marker of late-stage autophagy, was quantitated by flow cytometry and supravital staining with 1 µg/ml acridine orange (AO) (Sigma-Aldrich, St. Louis, MQ, USA). The exposed cells were harvested via the above-mentioned process and then stained with AO at 37 °C in the dark for 20 min. Green (500–550 nm, FL1 channel) and red (> 650 nm, FL3 channel) fluorescent signals, which were excited by blue light (488 nm), were measured by using a FACSCalibur flow cytometer equipped with the CellQuest software (BD Biosciences, Heidelberg, Germany). The ratio of FL3/FL1 (i.e., red-to-green fluorescence intensity) was considered to be an indicator of AVO formation.

### Cell cycle analysis

Cell cycle was evaluated by quantifying intracellular DNA using PI (Sigma-Aldrich, St. Louis, MQ, USA) through a flow cytometry-based approach. The exposed cells were trypsinized, washed with PBS, and centrifuged at 2000 rpm at 4 °C for 5 min. Afterwards, the pellets were resuspended in 500 µl PBS and then fixed by drop-wise addition of 2 ml pre-chilled 75% ethanol (cooled at − 20 °C in advance) at 4 °C overnight. The fixed cells were washed twice with PBS and subsequently incubated with PI (50 µg/ml) in the presence of RNase (100 µg/ml) (Sigma-Aldrich, St. Louis, MQ, USA) at 37 °C in the dark for 1 h. Finally, DNA contents of the stained cells were analyzed by a flow cytometer (BD Biosciences, Heidelberg, Germany). The histogram of cell cycle distribution was generated from 10,000 events per sample. The data were eventually presented as percentages of the cells in G0/G1, S, and G2/M phases using the CellQuest software (BD Biosciences, Heidelberg, Germany) and Modfit LT V5.0 (Verity Software House, Topsham, ME, USA).

### Western blot analysis

Total cellular proteins were extracted by lysis of the harvested cells with the protein extraction buffer at 4 °C for 1 h, followed by denaturation at 95 °C for 5 min, and then separated by regular sodium dodecyl sulfate (SDS)-polyacrylamide gel electrophoresis (PAGE) according to the procedure reported formerly [24]. Next, the proteins were transferred to nitrocellulose membranes (EMD Millipore, Billerica, MA, USA), after which the membrane was probed for the proteins of interest with specific antibodies. Finally, the immunoreactive bands were detected with an enhanced chemiluminescence (ECL) detection system (Amersham Life Sciences Inc., Arlington Heights, IL, USA). The expression of GAPDH was considered as a loading control. Primary and secondary antibodies used in this study includes: anti-LC3, anti-p62, anti-Beclin-1, anti-Bax, anti-Atg5, and anti-cyclin B (Cell Signaling Technology, Beverly, MA, USA); anti-p53, anti-PARP, anti-Caspase 3, anti-pro-Caspase 3, anti-cyclin A, and anti-GADPH (Santa Cruz Biotechnology, Santa Cruz, CA, USA); anti-p27, anti-CDK2, anti-mouse IgG, and anti-rabbit IgG (GeneTex, Irvine, CA, USA).

### In vivo exposure

All the experimental procedures were performed in accordance with the National Institutes of Health Guidelines for animal research (Guide for the Care and Use of Laboratory Animal) and approved by the National Cheng Kung University (NCKU) Institutional Animal Care and Use Committee (IACUC approval no. 104080). Adult female BALB/c mice (6–8 weeks of age, weighting 18–20 g) used in this study were purchased from Laboratory Animal Center of NCKU. All animals were pathogen free and housed in a controlled room (temperature 24 ± 2 °C, relative humidity 50 ± 10%, 12-h light/12-h dark cycle) with unrestricted access to food and water. To explore the manifestations of acute toxic effects exerted by AgNPs, mice were randomly assigned into one untreated control group (normal saline) and different selected doses of AgNPs (i.e., SCS and SAS particles, respectively)-treated groups (n = 3–5 per group and per time point). Normal saline was used as a diluent for injectable preparations of SCS and SAS suspensions, and each mouse of individual treatments was administered with a single dose of SCS (1, 2, 4, 6, and 8 mg/kg bw) and SAS (1, 8, 25 and 250 mg/kg bw), respectively via the IP route. The survival of the mice was determined at 2, 4, 7 and 14 days post-injection, and organ sampling was done right after survival observation.

### Statistical analysis

All in vitro experiments were conducted in triplicate and repeated three times, while all in vivo results, excepting those of ex vivo whole-organ IVIS imaging, were obtained by analyzing at least three independent samples. The quantitative data were expressed as mean ± standard deviation (SD) (in all Tables) or plotted by Sigma Plot v.12.0 (Systat Software Inc., San Jose, CA, USA). Statistical comparisons were performed using the two-tailed unpaired Student’s *t* test and one-way ANOVA. Statistical significance was defined as *P*-value of < 0.05.

## Supplementary Information

**Additional file 1: Fig. S1**. Physicochemical features of the four types of synthetic AgNPs (SCS, LCS, SAS and LAS). (A) Particle size and morphology, revealed by transmission electron microscopy (TEM) (Scale bar: 20 nm, for SCS and SAS; 100 nm, for LCS and LAS); (B) Chemical composition, analyzed by energy-dispersive X-ray (EDX) spectrometry; (C) and (D) Particle size distribution (in water- and medium-based suspensions, each), measured by the dynamic light scattering (DLS) method; (E) Absorbance spectrum (in water- or medium-based suspensions), determined by UV–Vis spectrophotometry. **Fig. S2** Single-cell-line-versus-four-AgNP-types dose–response patterns at 24 and 48 h post-exposure. (A) BEAS-2B; (B) Clone 9; (C) HaCaT; (D) HEK293; (E) THP-1; (F) IEC-6; and (G) AML12, which are reorganized from the cell viability results depicted in Figs. 1 and 2. *(*P* < 0.05), **(*P* < 0.01) and *** (*P* < 0.005) denote significant differences in cell viability between different particle type groups. **Fig. S3** The influence of exposure to SCS, LCS, SAS, or LAS on the statuses of cell death modalities (apoptosis, necrosis, and autophagy) occurring in IEC-6 cells. (A) and (B) Apoptotic and necrotic events in response to SAS exposure (1, 5, 10 and 15 µg/ml) for 24 h, as measured by flow cytometry using annexin V-FITC/propidium iodide (PI) staining (Annexin V-FITC positive/PI negative cells are those undergoing early-stage apoptosis, while annexin V-FITC positive/PI negative cells are those in the late stage of apoptosis. Necrotic cells are considered to be stained with PI alone. Total apoptotic cells = early apoptotic cells + late apoptotic cells); (C) Autophagic events in response to respective exposures to SCS, LCS, SAS, and LAS (5 µg/ml) for 8 h, as measured by flow cytometry with acridine orange (AO) staining. (D) Time-course analyses of the autophagic activity in response to serial doses of SAS (0.5, 1, 5, 10 and 15 µg/ml). Results were representative of three independent experiments performed in triplicate. *(*P* < 0.05), **(*P* < 0.01) and *** (*P* < 0.005) denote significant differences between the control and treatment groups. **Fig. S4** Cellular senescence and proliferative inhibition evoked by longer exposures to noncytotoxic doses of LCS particles. (A) Different degrees of senescence-associated β-galactosidase (SA-β-gal) activity in untreated control and LCS-treated AML12 cells (1, 5 and 10 µg/ml; 48 h post-treatment); (B) The capacity of AML12 cells to survive and proliferate following challenge with LCS (1 and 5 µg/ml) for 48 h, as evaluated by the clonogenic assay. (C) Quantification of the colony-forming capacity of untreated control and LCS-treated cells (indicated by “surviving fraction (SF)”; please refer to “clonogenic assay” in the following supplementary materials and methods). *(*P* < 0.05), **(*P* < 0.01) and *** (*P* < 0.005) denote significant differences between the control and treatment groups.

## Data Availability

The datasets used and/or analyzed during the current study are available from the corresponding authors upon a reasonable request.

## Abbreviations

AgNPs: Silver nanoparticles
ENMs: Engineered nanomaterials
NPs: Nanoparticles
ROS: Reactive oxygen species
KDD: Knowledge discovery in databases
DM: Data mining
PVP: Polyvinylpyrrolidone
SCS: Smaller citrate-coated AgNPs
LCS: Larger citrate-coated AgNPs
SAS: Smaller cysteamine-coated AgNPs
LAS: Larger cysteamine-coated AgNPs
TEM: Electron transmission microscopy
EDX: Energy-dispersive X-ray
DLS: Dynamic light scattering
PALS: Phase analysis light scattering
PDI: Polydispersity index
TP: True positive
FP: False positive
LDH: Lactate dehydrogenase
PI: Propidium iodide
SA-β-gal: Senescence-associated β-galactosidase
LC3: Microtubule-associated protein 1 light chain 3
PARP: Poly(adenosine diphosphate ribose) polymerase
CDKs: Cyclin-dependent kinases
IP: Intraperitoneal
RBITC: Rhodamine B isothiocyanate
ALT: Alanine aminotransferase
AST: Aspartate aminotransferase
CRE: Serum creatinine
BUN: Blood urea nitrogen
GLU: Glucose
ALY: Amylase
