# Supplementary material for: Use of an in silico knowledge discovery approach to determine mechanistic studies of silver nanoparticles-induced toxicity from in vitro to in vivo
Source: Part Fibre Toxicol. 2022 Jan 14;19:6. doi: 10.1186/s12989-022-00447-0 (PMC8759195; doi:10.1186/s12989-022-00447-0)
Supplement: Supplementary file 1 — Additional file 1: Fig. S1. Physicochemical features of the four types of synthetic AgNPs (SCS, LCS, SAS and LAS). (A) Particle size and morphology, revealed by transmission electron microscopy (TEM) (Scale bar: 20 nm, for SCS and SAS; 100 nm, for LCS and LAS); (B) Chemical composition, analyzed by energy-dispersive X-ray (EDX) spectrometry; (C) and (D) Particle size distribution (in water- and medium-based suspensions, each), measured by the dynamic light scattering (DLS) method; (E) Absorbance spectrum (in water- or medium-based suspensions), determined by UV–Vis spectrophotometry. Fig. S2 Single-cell-line-versus-four-AgNP-types dose–response patterns at 24 and 48 h post-exposure. (A) BEAS-2B; (B) Clone 9; (C) HaCaT; (D) HEK293; (E) THP-1; (F) IEC-6; and (G) AML12, which are reorganized from the cell viability results depicted in Figs. 1 and 2. *(P < 0.05), **(P < 0.01) and *** (P < 0.005) denote significant differences in cell viability between different particle type groups. Fig. S3 The influence of exposure to SCS, LCS, SAS, or LAS on the statuses of cell death modalities (apoptosis, necrosis, and autophagy) occurring in IEC-6 cells. (A) and (B) Apoptotic and necrotic events in response to SAS exposure (1, 5, 10 and 15 µg/ml) for 24 h, as measured by flow cytometry using annexin V-FITC/propidium iodide (PI) staining (Annexin V-FITC positive/PI negative cells are those undergoing early-stage apoptosis, while annexin V-FITC positive/PI negative cells are those in the late stage of apoptosis. Necrotic cells are considered to be stained with PI alone. Total apoptotic cells = early apoptotic cells + late apoptotic cells); (C) Autophagic events in response to respective exposures to SCS, LCS, SAS, and LAS (5 µg/ml) for 8 h, as measured by flow cytometry with acridine orange (AO) staining. (D) Time-course analyses of the autophagic activity in response to serial doses of SAS (0.5, 1, 5, 10 and 15 µg/ml). Results were representative of three independent experiments per [file 12989_2022_447_MOESM1_ESM.docx]

**Use of an *In silico* Knowledge Discovery Approach to Translate Mechanistic Studies of Silver Nanoparticles-induced Toxicity from *In vitro* to *In vivo***

Bin-Hsu Mao^1, #^, Yi-Kai Luo^1^, Bour-Jr Wang^2,3^, Fong-Yu Cheng^4^, Yu-Hsuan Lee^5, #^,

Shian-Jang Yan^6*^, and Ying-Jan Wang^1, 7**^

Supplementary file contains the following:

Supplementary figures 1-4

Supplementary table 1

Supplementary methods and their references

**
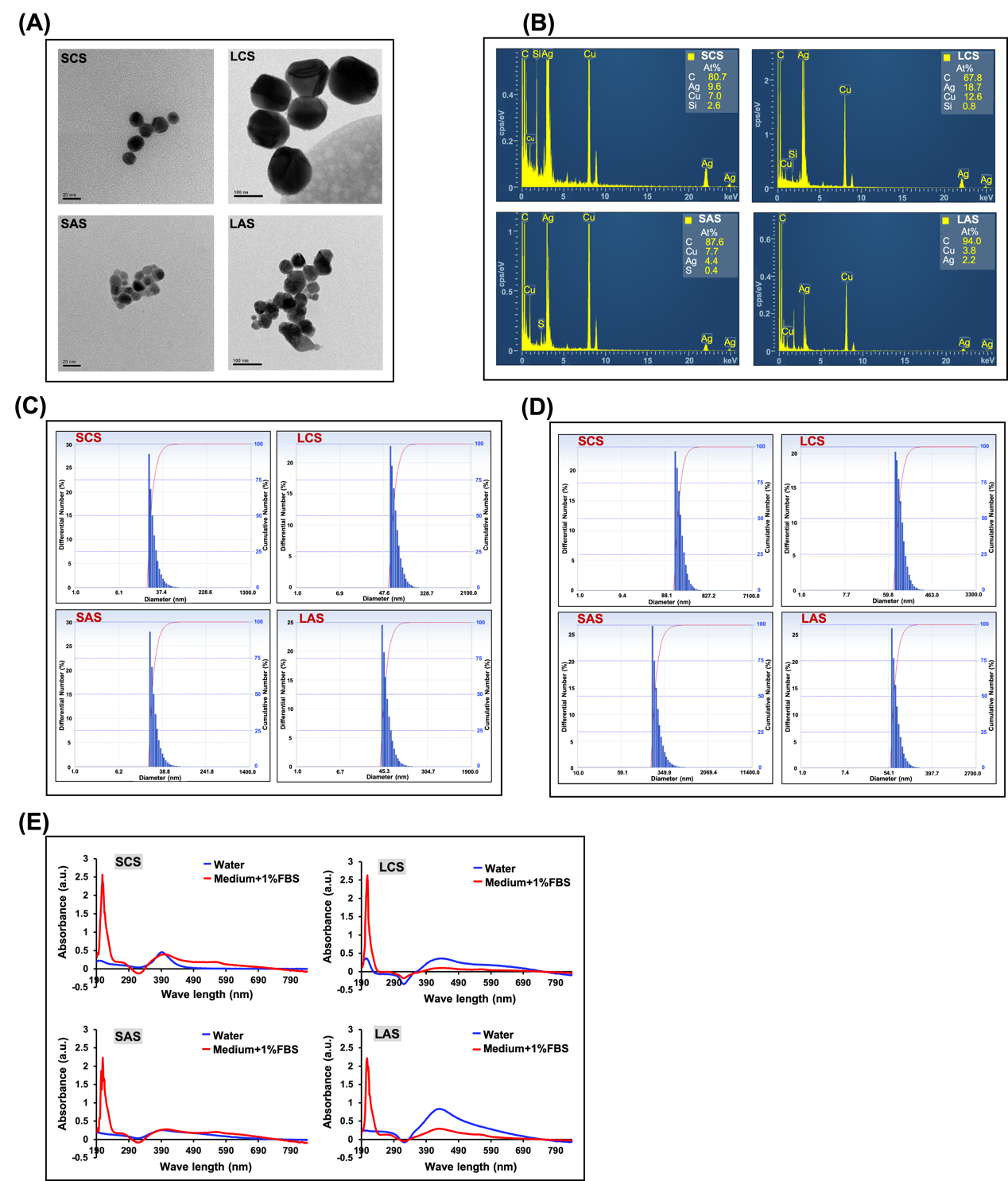
Supplementary figure 1**

**Physicochemical features of the four types of synthetic AgNPs (SCS, LCS, SAS and LAS).** (A) Particle size and morphology, revealed by transmission electron microscopy (TEM) (Scale bar: 20 nm, for SCS and SAS; 100 nm, for LCS and LAS); (B) Chemical composition, analyzed by energy-dispersive X-ray (EDX) spectrometry; (C) and (D) Particle size distribution (in water- and medium-based suspensions, each), measured by the dynamic light scattering (DLS) method; (E) Absorbance spectrum (in water- or medium-based suspensions), determined by UV-Vis spectrophotometry.

**
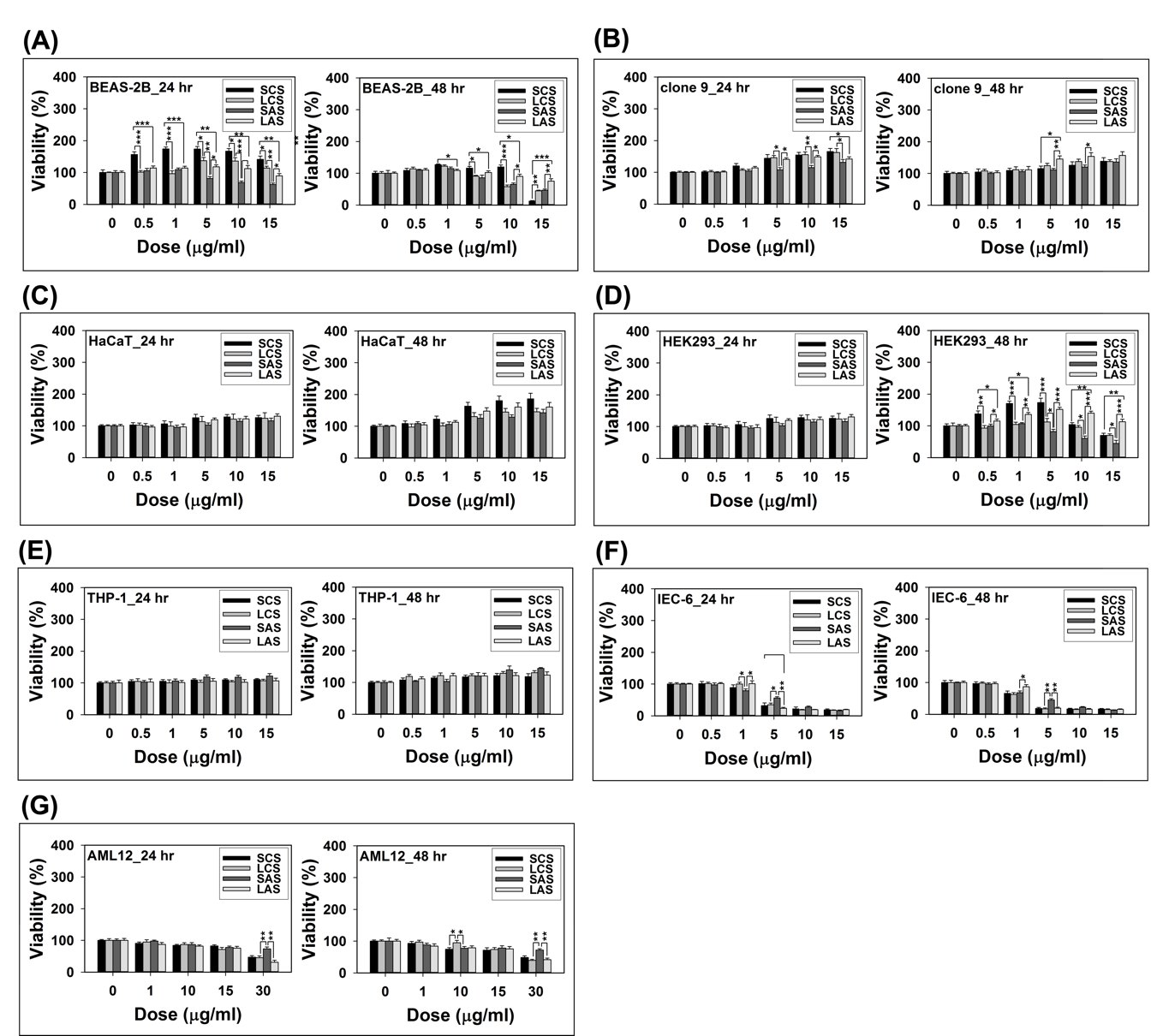
Supplementary figure 2**


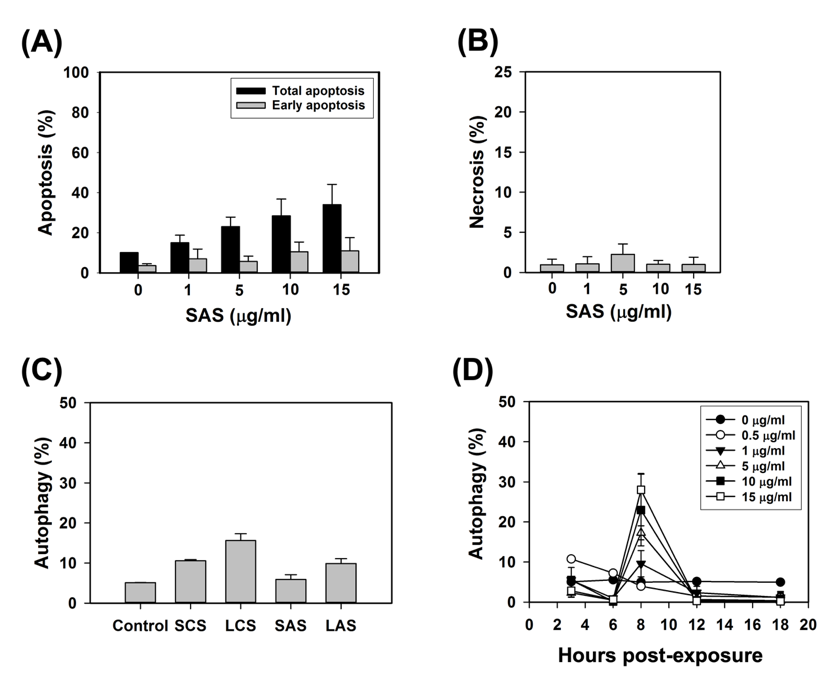


**Single-cell-line-versus-four-AgNP-types dose-response patterns at 24 and 48 hrs post-exposure.** (A) BEAS-2B; (B) Clone 9; (C) HaCaT; (D) HEK293; (E) THP-1; (F) IEC-6; and (G) AML12, which are reorganized from the cell viability results depicted in Figures 1 and 2. *(*P*<0.05), **(*P*<0.01) and *** (*P*<0.005) denote significant differences in cell viability between different particle type groups.

**
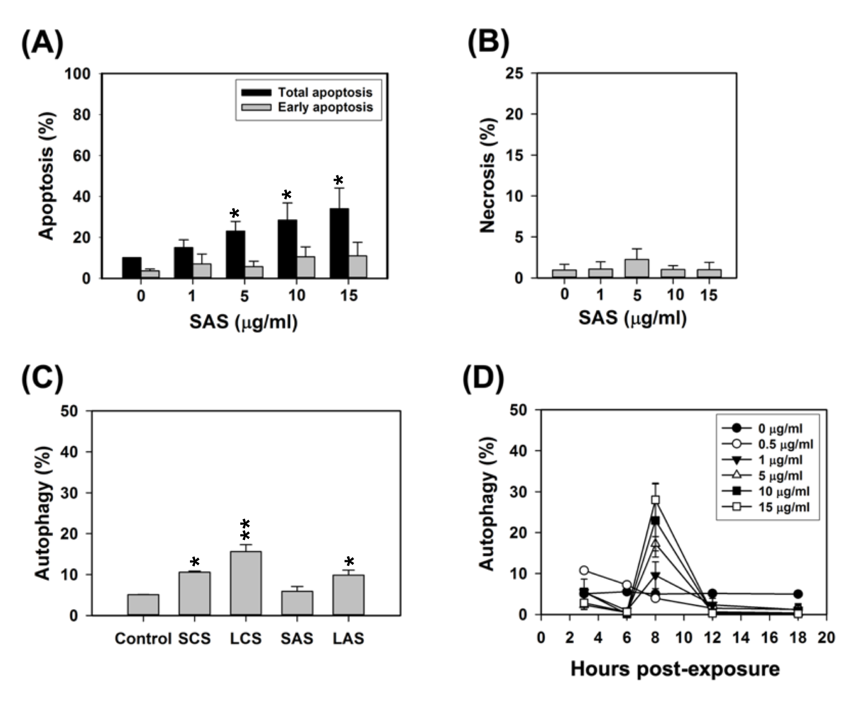
Supplementary figure 3**

**The influence of exposure to SCS, LCS, SAS, or LAS on the statuses of cell death modalities (apoptosis, necrosis, and autophagy) occurring in IEC-6 cells.** (A) and (B) Apoptotic and necrotic events in response to SAS exposure (1, 5, 10, and 15 μg/ml) for 24 hrs, as measured by flow cytometry using annexin V-FITC/propidium iodide (PI) staining (Annexin V-FITC positive/PI negative cells are those undergoing early-stage apoptosis, while annexin V-FITC positive/PI negative cells are those in the late stage of apoptosis. Necrotic cells are considered to be stained with PI alone. Total apoptotic cells = early apoptotic cells + late apoptotic cells); (C) Autophagic events in response to respective exposures to SCS, LCS, SAS, and LAS (5 μg/ml) for 8 hrs, as measured by flow cytometry with acridine orange (AO) staining. (D) Time-course analyses of the autophagic activity in response to serial doses of SAS (0.5, 1, 5, 10, and 15 μg/ml). Results were representative of three independent experiments performed in triplicate. *(*P*<0.05), **(*P*<0.01) and *** (*P*<0.005) denote significant differences between the control and treatment groups.

**Supplementary figure 4**

**
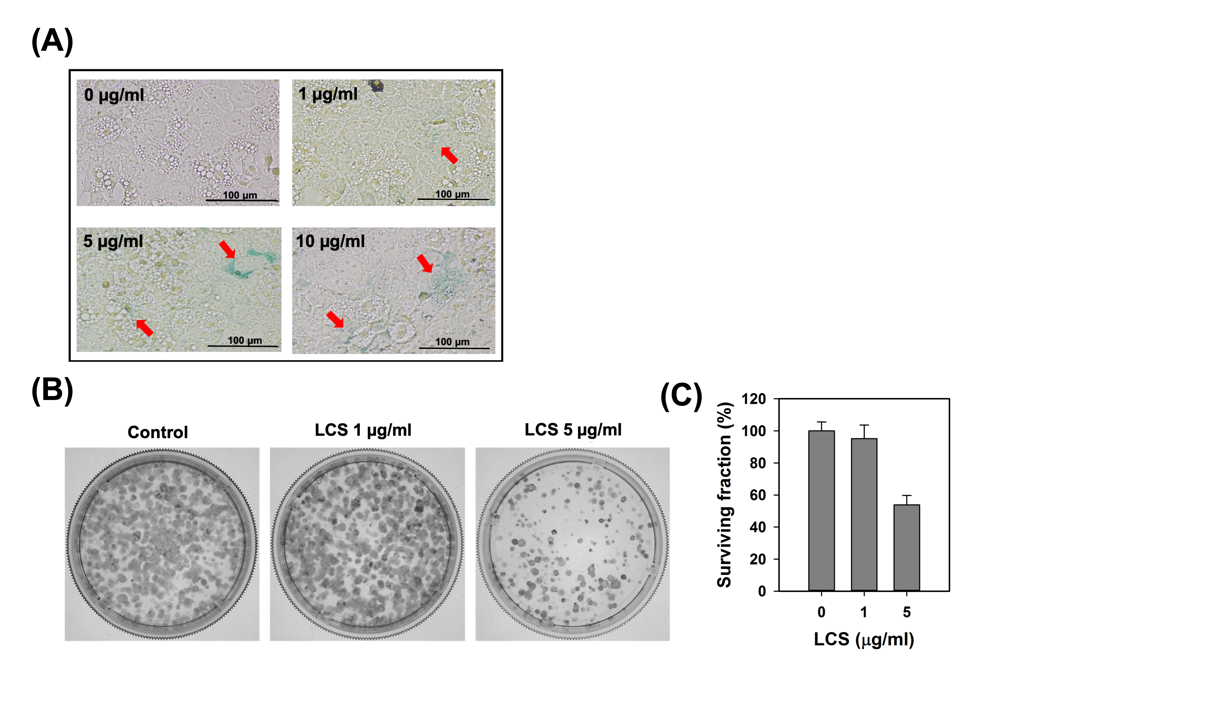
**

**Cellular senescence and proliferative inhibition evoked by longer exposures to noncytotoxic doses of LCS particles.** (A) Different degrees of senescence-associated β-galactosidase (SA-β-gal) activity in untreated control and LCS-treated AML12 cells (1, 5 and 10 μg/ml; 48 hrs post-treatment); (B) The capacity of AML12 cells to survive and proliferate following challenge with LCS (1 and 5 μg/ml) for 48 hrs, as evaluated by the clonogenic assay. (C) Quantification of the colony-forming capacity of untreated control and LCS-treated cells (indicated by “surviving fraction (SF)”; please refer to “clonogenic assay” in the following supplementary materials and methods). *(*P*<0.05), **(*P*<0.01) and *** (*P*<0.005) denote significant differences between the control and treatment groups.

**Supplementary table 1**

Table S1. Mammalian cell lines and the culture media used in this study.

| Cell line name | Source | Culture medium |
| --- | --- | --- |
| BEAS-2B cell | human lung epithelial cells | LHC-9 basal growth medium containing 0.1% 25 mM L-glutamine, 0.1% 1mM pyruvate, 0.1% non-essential 1 mM amino acids, 1% antibiotics, and 10% fetal bovine serum (FBS) |
| clone 9 | rat liver epithelial cells | DMEM F-12K containing 1% 25 mM L-glutamine, 1% antibiotics, and 10% FBS |
| HaCaT | human epidermal keratinocytes | DMEM containing 4 mM Ultra Glutamine I, 1% antibiotics, and 10% FBS |
| HEK-293 | human embryonic kidney cells | DMEM containing 1 mM non-essential amino acids, 1.0 mM sodium pyruvate, 1% antibiotics, and 10% Fetal bovine |
| IEC-6 | rat small intestinal epithelial cells | DMEM containing 4 mM L-glutamine, 1.0 mM sodium pyruvate, 0.1 Unit/ml bovine insulin, 1% antibiotics, and 5% FBS |
| THP-1 | human monocytes | RPMI medium containing 10.0 mM HEPES, 1.0 mM sodium pyruvate, 0.05 mM 2-ME, 1% antibiotics, and 10% FBS |
| AML 12 | mouse liver epithelial cells | DMEM F-12 containing 0.005 mg/ml insulin, 0.005 mg/ml transferrin, 5 ng/ml selenium, 40 ng/ml dexamethasone, 1% antibiotics, and 10% FBS |

**Supplementary methods and their references**

**Fabrication and characterization of SCS, LCS, SAS and LAS particles**

Syntheses of smaller- and larger-sized citrate-coated AgNPs (i.e., SCS and LCS) were carried out according to the formerly reported chemical reduction protocols of Solomon *et al.* and Šileikaitė *et al.*, respectively [1, 2]. In brief, SCS was prepared by dropwisely and slowly titrating a pyogen-free aqueous mixture (588 ml) containing 1 mM silver nitrate (AgNO_3_) and 3.2 mM trisodium citrate (Na_3_C_6_H_5_O_7_) with 12 ml of 100 mM ice-cold sodium borohydride (NaBH_4_) solution. The entire titration process was performed at 4°C under the agitation speed of 800 rpm for approximately 3 mins, and afterwards further agitation continued for another 2 hours. The resultant reddish brown SCS suspension was subjected to two-step centrifugation-based concentration and size refinement: larger agglomerates and objects in the colloidal suspension were removed in the first centrifugation process (7,000 xg, 30 mins), and then a concentrated colloidal dispersion of dimensionally homogeneous SCS particles was acquired in the second centrifugation (27,000 xg, 30 mins) by collecting the suspended pellet fraction.

As for LCS particles, 40 ml of 1 mM AgNO_3_ solution was heated to boiling in a round bottom flask equipped with a reflux condenser, submerged in an oil bath (120°C) on a magnetic hotplate stirrer. 0.5 ml of 1% (w/v) Na_3_C_6_H_5_O_7_ solution was added into the vigorously agitated AgNO_3_ solution drop by drop. In order to achieve a thorough reduction of silver ions, the reaction product remained in a boiling and stirring state for 30 mins. After ambient cooling, the visible granules in the suspension was removed by filtrating through filter paper. The filtrated suspension then underwent concentration and size refinement via centrifugation (5,000 xg, 30 mins). In the end, the concentrated pellet fraction was directly resuspended to obtain a colloidal dispersion of LCS particles.

Smaller and larger cysteamine (C_2_H_7_NS)-coated AgNPs (i.e., SAS and LAS) were fabricated by respectively replacing citrate ions from the surface of as-prepared SCS and LCS particles with cysteamine molecules, as previously reported [3]. Briefly, 40 ml of SCS suspension (40 μg/ml) was mixed with 100 μl of cysteamine solutions (100 mM), while the released hydrogen ions were neutralized by addition of 10 μl of NaOH (10 M). The reaction mixture was vigorously stirred at room temperature for 2 hrs, and then underwent centrifugation (27,000 xg, 30 mins). At last, the pellet fraction was collected and directly resuspended to obtain a SAS aqueous dispersion. The procedure for fabricating LAS particles was quite similar to that involved in SAS synthesis, with adjustments in concentration of cysteamine (500 mM), volume of NaOH (50 μl), and centrifugal speed (7,000 xg). After fabrication, all of the above-mentioned colloidal AgNP aqueous dispersions were stored at 4°C and kept from light.

The concentration of each AgNP sample was determined by graphite furnace atomic absorption spectrometry (GFAAS) (AAnalyst^TM^ 600, PerkinElmer, CA, USA). For analyzing the diameter, morphology, and chemical composition of these nanoparticles, transmission electron microscope (TEM) (JEM-2100F, JEOL, Tokyo, Japan) coupled with energy-dispersive X-ray spectrometer (EDX) (JED-2300T, JEOL, Tokyo, Japan) was used. The hydrodynamic size plus polydispersity index (PDI) of the dispersions were detected according to the dynamic light scattering (DLS) method, while the zeta potential was evaluated using the phase analysis light scattering (PALS) technique. These analyses were performed with the help of a nanoparticle size and zeta potential analyzer (Delsa^TM^ NanoC, Beckman Coulter, CA, USA). The absorption peaks of these nanosuspensions were measured with a UV-Vis spectrophotometer (NanoDrop 2000, Thermo Scientific, NY, USA).

**Preparation of smaller-sized RBITC-conjugated AgNP particles**

A volume of 20 ml of SAS aqueous dispersion (800 μg/ml) was incubated with 2 ml of 1 mM rhodamine B isothiocyanate (RBITC) (Sigma-Aldrich, St. Louis, MO, USA) under vigorous agitation at room temperature for 2 hrs. After the labeling reaction, the resulting product was subjected to centrifugal concentration (27,000 xg, 30 mins). The pellet fraction should be resuspended in pyogen-free water and then underwent repeated centrifugation until the supernatant fraction appeared colorless. Subsequently, the pellet fraction was directly resuspended to get a concentrated colloidal dispersion of smaller-sized RBITC-conjugated AgNPs.

***In vitro* AgNPs exposure**

Different concentrations of the working AgNPs suspensions (including SCS, LCS, SAS, and LAS), which were 50 times the ultimate exposure doses used in the *in vitro* experiments, were prepared in advance. Then, the culture medium containing 10% FBS was blended with the working suspension of a given concentration in a fixed volume ratio (49:1), which thus reduces alteration in proportions of the medium components. Redispersion of AgNPs in the culture medium was done in an ultrasonic bath (40khz, 500W) for 5 mins immediately preceding the exposure treatment. As for each separate experiment, cells from a single passage were subjected to the assessment to minimize confounding arising from passage-to-passage variations of the cultured cells. Results were replicated by independently conducting the experiments on cells of the identical passage.

**Cell viability assessment**

Two analogous cell viability assays performed in 96-well microplates were used for assessing cytotoxicity of the four AgNP types separately towards the abovementioned cell lines: MTS (for BEAS-2B, clone 9, HaCaT, HEK293, THP-1, and IEC-6) and MTT (for AML12). They both are based on reduction of the tetrazolium salts to chromogenic formazan products by mitochondrial dehydrogenases in metabolically active cells. The quantity of formazan is indicative of the number of viable cells in the culture and can be determined spectrophotometrically (for MTT: the absorbance is measured at 570 nm; for MTS: at 490 nm).

To account for any possible absorbance by AgNPs, a cell-free control was prepared for each concentration of the treatments. Briefly, the medium in wells with and without cells, at 24 hrs post-seeding, was replaced with the medium containing indicated doses of the particles (100 μl/well). After 24- and 48-hrs of incubation, the supernatants were removed, followed by addition of 100 μl of fresh phenol-red free medium and either 20 μl of MTS/CellTiter96^®^Aqueous One Solution Reagent (Promega, Madison, WI, USA) or 25 μl of MTT solution (5 mg/ml) (Sigma-Aldrich, St. Louis, MQ, USA) to each well. The reactions were allowed to develop for 3-4 hrs in the cell culture incubator. The MTS formazan product needed no further processing before reading, whereas the MTT product required a solubilization step with dimethyl sulfoxide (DMSO). Absorbance was measured using a SPECTRAmax M2 plate reader (Molecular Devices, Sunnyvale, CA, USA). All data were normalized by subtracting the blank absorbance values (in the absence of cells). Relative cell viability was calculated as follows: (A_treatment_-A_blank_) /(A_control_-A_blank_).

**Decision tree-based KDD process**

*In silico* prediction of AgNPs cytotoxicity was performed using the decision tree-based “Knowledge Discovery in Databases (KDD)” approach reported previously [4]. Briefly, the results of the cell viability assessments, acquired using either the MTS or MTT assay, were compiled with the toxicity-relevant attributes, comprising cell type, AgNP type, exposure dose, and exposure time, into a dataset. To execute data mining, three widely accepted toxicity threshold values (i.e., cell viability reductions by 20%, 25%, and 30%) were respectively used as the cut-off to convert the numeric viability results into binary nominal data (i.e., with/without toxicity). Note that in this research, the pro-proliferative response was considered non-toxic. The Waikato Environment for Knowledge Analysis (WEKA) software, developed at the University of Waikato, New Zealand, was used to classify the dependent variables through independent attributes, followed by choosing the decision trees (i.e., J48 algorithm) to display and interpret the relationship between attributes.

**Senescence-associated β-galactosidase activity assay**

Analysis of senescence-associated β-galactosidase activity (SA-β-gal) was carried out according to Dimri *et al* [5]. Briefly, after 48 hrs of AgNPs exposure, the cells were fixed with fixation solution (2% formaldehyde, 0.2% glutaraldehyde in PBS) at RT for 3-5 mins, washed with PBS, and then incubated in an oven at 37°C overnight with a mix solution of 1 mg/ml 5-bromo-4-chloro-3-indolyl-β-D-galactopyranoside (X-gal), 5 mM potassium ferrocyanide, 5 mM potassium ferricyanide, 150 mM NaCl, 2mM MgCl_2_, and 0.1 M phosphate buffer, pH 6.0. After incubation, the stained cells were washed twice with PBS, rinsed with methanol, air-dried, and eventually analyzed with an Olympus BX71 light inverted microscope equipped with a DP72 CCD camera.

**Clonogenic assay**

The influence of LCS particles on the colony-forming behavior of AML12 cells was determined using the method adapted from one previously described by Herzog et al. [6]. Briefly, cells were harvested from log-phase growing cultures and seeded at a density of 500 cells/well in a six-well microplate. After 24 hrs, cells were exposed to 1 and 5 μg/ml of LCS particles respectively for 48 hrs, followed by replacement of the test media with normal growth media (without containing LCS particles), and then kept in culture for additional 7 days. Afterwards, the media were removed, and the adherent colonies were fixed and stained with methanol containing 0.1% (w/v) Coomassie Blue (0.1% (w/v) Brilliant blue dye (Sigma-Aldrich, St. Louis, MQ, USA) in 10% (v/v) acetic acid and 90% (v/v) methanol) at RT for 5 mins. The stain was aspirated from the wells, and the stained colonies became evident by washing them with destain buffer (10% (v/v) acetic acid, 30% (v/v) methanol, and 60% (v/v) water). The plates were photographed with a digital camera, and the number of colonies was quantified with the Image-Pro Plus software program (Media Cybernetics, Silver Spring, MD, USA).

**Serum biochemistry analysis**

Blood specimens of the untreated controls and AgNPs-treated mice (SCS: 8 mg/kg, SAS: 8 mg/kg) (n=3 per group) were respectively drawn into serum-separating tubes through the venipuncture method. Afterwards, serum was obtained by allowing blood to clot at RT for 20-30 mins and then centrifuging it at 15,000 rpm at 4°C for 15 mins. The levels of serum biochemical parameters, including ALT, AST, CRE, BUN, GLU, and AMYL, were assessed by an automated blood biochemical analyzer, DRI-CHEM 3500s (Fujifilm, Kanagawa, Japan).

**Biodistribution and bioaccumulation assessments**

Smaller-sized RBITC-AgNPs at a dose of 8 mg/kg were intraperitoneally injected into the mice. At 1, 5, 24 and 48 hrs post-injection, mice (n=1 per time point) were respectively sacrificed and organs of interest (heart, lung, liver, spleen, and kidney) were collected for *ex vivo* whole-organ imaging analysis as previously described [7]. Macroscopic fluorescence images acquired under the IVIS Imaging System (Perkin Elmer, Waltham, MA, USA) were analyzed using the Living Image 4.7.2. software, where the ROIs were drawn for each organ to obtain the individual fluorescence signals.

To quantify visceral Ag accumulation, metabolic organs (liver, spleen, and kidney) of the AgNPs-treated mice and untreated counterparts were digested with a mixture of concentrated HNO_3_ and HCl (9:1) in a MARSXpress microwave-accelerated reaction system (MARS, CEM, Matthews, NC, USA) through the processes reported before [8]. The digested samples were diluted with 1% (v/v) HNO_3_ to desired final concentrations (approximately 500- to 1000-fold dilutions depending on the exposure dose) in advance of quantification using a graphite furnace atomic absorption spectrometer (AAnalyst^TM^ 600, PerkinElmer, CA, USA). Ag concentration was normalized to the weight of the digested organs for each measurement.

**Macroscopic and histopathological examinations**

Images of gross anatomy of the key metabolic organs (liver, spleen, pancreas and kidney) was documented using a Leica stereomicroscope MZ10F equipped with a digital camera (Leica Inc., Wetzlar, Germany). For histopathological examination, the fixation, dehydration, clearing, impregnation, paraffin embedding and blocking, section cutting, and Hematoxylin & Eosin (H&E) staining processes of the liver and spleen specimens were conducted as formerly mentioned [9]. Finally, the mounted specimens were observed by standard bright field microscopy (Eclipse Ci-L, Nikon Instruments Inc., Japan).

**References**

1. Solomon, S. D.; Bahadory, M.; Jeyarajasingam, A. V.; Rutkowsky, S. A.; Boritz, C.; Mulfinger, L., Synthesis and study of silver nanoparticles. *J Chem Educ* **2007,** 84, (2), 322-325.

2. Šileikaitė, A.; Puišo, J.; Prosyčevas, I.; Tamulevičius, S., Investigation of silver nanoparticles formation kinetics during reduction of silver nitrate with sodium citrate. *Materials Science (Medžiagotyra)* **2009,** 15, (1), 21-27.

3. Chen, Z. Y.; Li, N. J.; Cheng, F. Y.; Hsueh, J. F.; Huang, C. C.; Lu, F. I.; Fu, T. F.; Yan, S. J.; Lee, Y. H.; Wang, Y. J., The Effect of the Chorion on Size-Dependent Acute Toxicity and Underlying Mechanisms of Amine-Modified Silver Nanoparticles in Zebrafish Embryos. *Int J Mol Sci* **2020,** 21, (8).

4. Horev-Azaria, L.; Baldi, G.; Beno, D.; Bonacchi, D.; Golla-Schindler, U.; Kirkpatrick, J. C.; Kolle, S.; Landsiedel, R.; Maimon, O.; Marche, P. N.; Ponti, J.; Romano, R.; Rossi, F.; Sommer, D.; Uboldi, C.; Unger, R. E.; Villiers, C.; Korenstein, R., Predictive toxicology of cobalt ferrite nanoparticles: comparative in-vitro study of different cellular models using methods of knowledge discovery from data. *Part Fibre Toxicol* **2013,** 10, 32.

5. Dimri, G. P.; Lee, X.; Basile, G.; Acosta, M.; Scott, G.; Roskelley, C.; Medrano, E. E.; Linskens, M.; Rubelj, I.; Pereira-Smith, O.; et al., A biomarker that identifies senescent human cells in culture and in aging skin in vivo. *Proc Natl Acad Sci U S A* **1995,** 92, (20), 9363-7.

6. Herzog, E.; Casey, A.; Lyng, F. M.; Chambers, G.; Byrne, H. J.; Davoren, M., A new approach to the toxicity testing of carbon-based nanomaterials--the clonogenic assay. *Toxicol Lett* **2007,** 174, (1-3), 49-60.

7. McGowan, J. W.; Bidwell, G. L., 3rd, The Use of Ex Vivo Whole-organ Imaging and Quantitative Tissue Histology to Determine the Bio-distribution of Fluorescently Labeled Molecules. *J Vis Exp* **2016**, (118).

8. Wu, M.; Chen, L.; Li, R.; Dan, M.; Liu, H.; Wang, X.; Wu, X.; Liu, Y.; Xu, L.; Xie, L., Bio-distribution and bio-availability of silver and gold in rat tissues with silver/gold nanorod administration. *RSC advances* **2018,** 8, (22), 12260-12268.

9. Ibrahim, K. E.; Al-Mutary, M. G.; Bakhiet, A. O.; Khan, H. A., Histopathology of the Liver, Kidney, and Spleen of Mice Exposed to Gold Nanoparticles. *Molecules* **2018,** 23, (8).
